# Morphological Degradation of Oxygen Evolution Reaction-Electrocatalyzing Nickel Selenides at Industrially Relevant Current Densities

**DOI:** 10.1021/acsami.5c05381

**Published:** 2025-07-11

**Authors:** Felix Hiege, Chun-Wai Chang, Oliver Trost, Charlotte E. R. van Halteren, Pouya Hosseini, Georg Bendt, Stephan Schulz, Zhenxing Feng, Julia Linnemann, Kristina Tschulik

**Affiliations:** † Faculty of Chemistry and Biochemistry, Chair of Analytical Chemistry II, 9142 Ruhr University Bochum, Bochum 44801, Germany; ‡ School of Chemical, Biological, and Environmental Engineering, 2694 Oregon State University, Corvallis, Oregon 97331, United States; § Faculty of Chemistry, Chair of Inorganic Chemistry, and Center for Nanointegration Duisburg-Essen (Cenide), 27170 University Duisburg-Essen, Essen 45141, Germany; ∥ Max-Planck-Institut für Nachhaltige Materialien GmbH, Max-Planck-Straße 1, Düsseldorf 40237, Germany

**Keywords:** oxygen evolution reaction (OER), ultramicroelectrode (UME), Ni–Se, electrodeposition, electrochemical impedance spectroscopy (EIS), alkaline electrolytes

## Abstract

We investigated electrodeposited nanoparticulate nickel selenide (pre)­catalysts that transform into nickel oxides/oxyhydroxides under oxygen evolution reaction conditions in alkaline solutions. Previous studies of this transformation were conducted at lower current densities than those of industrial relevance (≥1 A cm^–2^). We used ultramicroelectrodes (UMEs) to achieve such current densities, benefiting from their small size, ensuring low absolute currents and low ohmic drop but high current densities. Morphological degradation of the catalyst material was only observed at current densities exceeding 1 A cm^–2^ but not for smaller ones. Using X-ray absorption, X-ray photoemission spectroscopy, and X-ray diffraction, we confirmed that the degradation was accompanied by the literature-known transformation of nanoparticulate Ni_3_Se_2_ (bulk)/NiSe (surface) into nickel oxyhydroxide. The transformation of the precatalyst goes along with a significant improvement in the charge transfer kinetics observed by decreasing Tafel slopes with ongoing experimental time extracted from cyclic voltammetry (CV) experiments and electrochemical impedance spectroscopy (EIS) in the high-frequency range. However, these kinetic improvements are accompanied by limitations in mass transport concluded from decreasing current responses at high overpotentials in CVs and increasing impedance in the low-frequency range of the EIS spectra after extended CV cycling. These mass transport limitations originated from morphological degradations at the UME exceeding 1 A cm^–2^ which we proved by applying identical location scanning electron microscopy. This has not been reported in studies that have been limited to lower current densities before. Our findings showcase how UMEs can be used to study (pre)­catalysts (herein nickel selenides) under current densities of industrial relevance in the absence of ohmic drop-related ambiguities, combined with in-depth materials characterization studies, e.g., identical location microscopy and advanced spectroscopic methods. This approach enables direct evaluation and comparison of catalyst materials and thus demonstrates how to overcome long-standing limitations of electrocatalyst design and testing.

## Graphical abstract

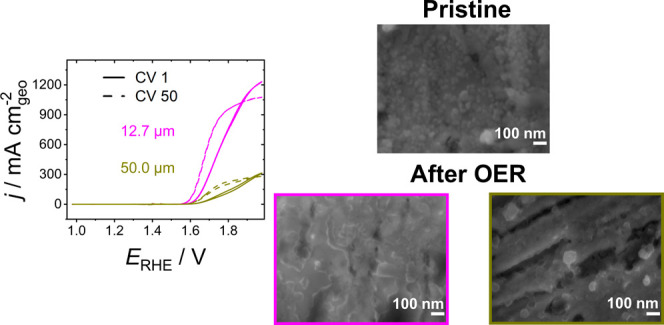

## 1 Introduction

Electrochemical water splitting to store energy as hydrogen is being discussed as one of the key technologies in the transition to renewable energy. Corresponding alkaline electrolyzers are mostly based on the kinetically sluggish oxygen evolution reaction (OER) as an anodic half-reaction and therefore use metal oxide catalysts.^1,2^ Much research is currently underway to develop stable non-noble metal-based electrocatalysts with comparable performance to noble metal oxides such as RuO_2_ and IrO_2_.^3,4^ Interestingly, some metal chalcogenide nanomaterials other than oxides were found to have improved electrocatalytic performance for alkaline OER, despite being converted to oxide and oxyhydroxide species in the interphase region with the electrolyte under OER conditions.^5–8^ Among these, nickel selenides as precatalysts that transform at anodic potentials in aqueous electrolytes have attracted considerable attention due to the observed electrocatalytic performance.^6,9,10^ Recent research suggests that selenium is replaced by oxygen at the surface and within the uppermost catalyst layers during anodic potential sweeps, leading to the formation of surface-near nickel oxide and oxyhydroxide-based structures.^5,7,9,11^ Several studies have shown that these anodically generated nickel oxide/oxyhydroxide species on nickel selenide templates show higher electrocatalytic performance than corresponding electrodeposited nickel oxide and oxyhydroxide catalysts.^5,9,11,12^ Noteworthy, the performance of all nickel oxide- and oxyhydroxide-based OER electrocatalysts depends strongly on the accessibility of iron ions in the electrolyte. This applies to both, nickel (oxyhydr)­oxide deposited directly or formed by anodic transformation of a precatalyst species, and is described in the literature as the “iron effect”.^13–20^ Kuai et al.^14,15^ attributed this well-known behavior to the incorporation of iron into the (surface) lattice following a dissolution/redeposition mechanism of iron ions. Depending on the specific nickel selenide precatalyst, differences in the resulting electrocatalytic activity have been observed.

Wu et al. identified Ni_3_Se_2_ as catalytically most active species compared to, e.g., NiSe.^7^ The reasons for the improved electrocatalytic activity of partially converted nickel selenides compared with directly synthesized nickel oxide and hydroxide catalysts are still under debate. Current hypotheses include a higher degree of disorder in the crystal structure, e.g., by irregular bond lengths between transition-metal centers and chalcogenide ions or vacancies in the crystal lattice,^5,7,12^ leading to an optimized ratio of exposed active sites, and increased electron mobility or facilitation of surface oxidation due to changes in the electronic band structure.^21,22^ However, characterization of the catalytically active site and its transformation under reaction conditions require operando investigations of nickel selenide-based materials using spectroscopic (e.g., X-ray absorption spectroscopy (XAS)) or microscopic (e.g., transmission electron microscopy (TEM)) techniques coupled with electrochemical techniques recording data under OER conditions.^23^

In this study, we aim to investigate the changes in nickel selenides during the alkaline OER at high current densities and relate them to the development of electrocatalytic activity. In laboratory scale studies, commonly used rotating disc electrode (RDE) approaches, also used in previous studies of nickel selenide OER electrocatalysts,^5–7,12^ are usually operated well below 100 mA cm^–2^.^9,13,24^ In linear potential sweep measurements, catalyst-coated RDEs typically allow for sufficient mass transport to achieve a potential region of purely kinetically controlled current which is suitable for physical electrochemical studies, including Tafel analysis.^24^ However, to enable an economically viable production of hydrogen via water electrolysis at the cathode side, OER current densities of >1 A cm^–2^ are commonly required at the anode.^25,26^ Thus, we used microelectrodes with electrodeposited nickel selenides to investigate the structural and catalytic development of nickel selenides at industrially relevant current densities (>1 A cm^–2^). This allows for higher current densities than those with RDEs but still enables in-depth electrochemical investigations of materials where currents are not affected by the complexity of an electrolyzer device.

For example, Bandarenka et al. used Pt-ultramicroelectrodes (UMEs) in the past to study the OER activity of metal oxide-based catalyst materials under high current density conditions.^27,28^ The approach takes advantage of the convergent diffusion mode present at microelectrodes, where the scale of the disc electrode becomes negligible compared to that of the growing diffusion layer in front of the electrode. The effect is shown schematically in Figure 1.

**Figure 1:**
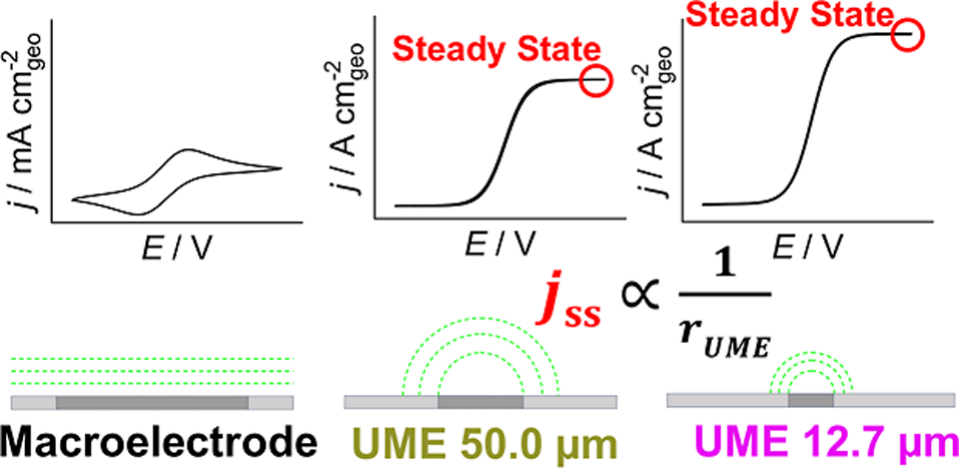
Schematic cyclic voltammograms (top) and corresponding schematic diffusion profiles (bottom) of an electrochemically active redox couple investigated with a macroelectrode (left), an UME with a diameter of 50.0 μm (middle) and an UME with a diameter of 12.7 μm (right).

In the absence of convection (e.g., stirring or electrode rotation), linear diffusion is observed at planar macroelectrodes. This results in a “classical” cyclic voltammogram with current peaks (for an electrochemical outer-sphere reaction).^29,30^ After reaching a maximum current, the diffusion-controlled current decreases as the diffusion layer thickness increases during the potential sweep. In contrast, cyclic voltammograms of UMEs exhibit a sigmoidal shape due to the spherical expansion of the diffusion layer, where the area of planes of equal concentration increases.^29,30^ At steady state, the diffusion flux becomes independent of time, and the mass-transport-limited current is constant. The smaller the microelectrode radius *r*_UME_ is, the higher the steady-state current density *j*_SS_ that can be achieved.^29,30^ This allows very high current densities in electrocatalytic studies using microelectrodes. The approach also often widens the potential window suitable for extracting kinetic parameters thanks to the efficient mass transport regime.^24^ Simultaneously, nominal currents decrease with the electrode area. Therefore, the influence of the system’s ohmic potential drop (*iR*-drop) is small, making *iR*-correction obsolete.^29–31^

Besides UMEs, further electrode setups providing enhanced mass transport conditions were used in literature reports investigating electrocatalytic reactions. For example, flow cell setups^32,33^ —which could even be coupled to electrolyzer stacks^34^—or membrane electrode assemblies (MEAs)^35^ allow OER current densities of several hundreds of mA cm^–2^ to A cm^–2^ scale. The evolving gas at the electrodes (oxygen at the anode, hydrogen at the cathode) is removed from the electrode surface by applying pressure on the backside of the permeable electrode support.^32–35^ However, those macro electrode setups still require ohmic drop correction (which can also be performed automatically by some potentiostat systems during data recording).^36^ Nanoelectrodes (*d* ≪ 1 μm) on the other hand theoretically provide due to their smaller nanometer-dimension a (hemi)­spherical diffusion profile conditions with lower mass transport limitations than UMEs.^37,38^ The absolute current is in the range of pA to nA, whereas the current density can exceed >1 A cm^–2^. Nanoelectrodes have been used in recent years to study electrocatalytic reactions, e.g., OER, on single nanoparticles to gain mechanistic insights of the investigated catalyst system.^39^ Noteworthy, these electrode geometries, flow cells, GDEs, MEAs, and nanoelectrodes are much more complicated to set up and control under catalytic conditions than UMEs.

In this study, we have electrodeposited nanoparticulate nickel selenide films onto UMEs of 12.7 and 50.0 μm diameter. This allowed us to investigate the influence of enhanced mass transport and thus high current densities in a less complex setup compared to alternative geometries. Our electrochemical protocol was tailored to study the activation and degradation of the (pre)­catalyst deposits (see Supporting Information experimental section for details). To the best of our knowledge, we found for the first time a morphological degradation, which occurred mainly on the 12.7 μm microelectrode and was caused by the high mass flux associated with the current density of >1 A cm^–2^ obtained at this electrode.

## 2 Experimental Section

Detailed information on the preparation of electrodes, electrodeposition, and measurement protocols as well as employed devices can be found in the Supporting Information.

### 2.1 Electrodeposition of Nickel Selenides

To investigate the OER-electrocatalytic activity of nickel selenides and its development under conditions of high current densities, nickel selenide was electrodeposited under coulometric control on gold microelectrodes (12.7 and 50.0 μm in diameter) using a three-electrode setup. The electrodeposition followed the method previously reported by Cao et al.^8^ from a deposition bath containing 10 mM NiSO_4_, 10 mM SeO_2_, and 25 mM K_2_SO_4_ at −0.79 V vs Ag|AgCl|3 M KCl on Au support material. Cao et al. reported the bulk structure of the electrodeposit as Ni_3_Se_2_.^8^

### 2.2 Electrochemical Investigation of Oxygen Evolution Reaction—Electrocatalytic Activity, Activation, and Degradation in Alkaline Media

Electrochemical investigations under OER conditions were performed using a three-electrode setup in 0.1 M KOH with 1 mM Fe­(NO_3_)_3_.^17^ A defined amount of Fe­(III) was added to the alkaline electrolyte to ensure experimental reproducibility as the activity of nickel chalcogenide OER electrocatalysts in alkaline media is known to depend on the availability of iron ions in the electrolyte (see Section 1).^13–20^ UMEs of different diameters were used to investigate the OER activity of the electrodeposited films under varying mass transport conditions. The full electrochemical protocol is detailed in Supporting Information-Section 1.4. Tafel slopes and overpotential trends were derived from two 25-cyclic voltammetry (CV)-cycle experiments at 10 mV s^–1^. Electrochemical impedance spectroscopy (EIS) measurements at 1.8 V vs RHE were carried out before, between, and after the two 25-CV-cycle periods and analyzed to follow the catalyst’s activation and degradation under OER conditions in terms of charge transfer (higher-frequency regions) and mass transport (lower-frequency regions).

### 2.3 Morphological, Structural, and Compositional Characterization of Electrodeposited Nickel Selenide Electrocatalysts

Morphological, structural, and compositional characterizations of the electrodeposited nickel selenides on macro electrodes were conducted using scanning electron microscopy (SEM) with energy-dispersive X-ray spectroscopy (EDX) (Supporting Information-Section 1.5.1), XAS (Supporting Information-Section 1.5.4), and X-ray photoelectron spectroscopy (XPS) (Supporting Information-Section 1.5.5).

Identical location SEM/EDX studies were performed on UMEs of both diameters (12.7 and 50.0 μm; Figure S17) to assess the composition and morphology of both the pristine nickel selenides and the transformed material after undergoing the full electrochemical protocol (denoted as “after OER”).

## 3 Results and Discussion

### 3.1 Structural, Morphological, and Compositional Analyses of Electrodeposited Nickel Selenides

To investigate the electrocatalytic activity of nanoparticular nickel selenides, electrodeposition from an aqueous precursor solution containing equimolar amounts of NiSO_4_ and SeO_2_ following the procedure reported by Cao et al.^8^ was performed on gold micro- and macroelectrodes. As shown in Figure 2a, as-deposited pristine films were subjected to electrochemical conditioning in alkaline electrolytes throughout ten CV cycles. This procedure activated the catalyst film, as indicated by the increasing current of the nickel redox wave and the decreasing OER overpotential. It also ensures consistency between similarly prepared samples undergoing different parts of the experimental framework (different electrode sizes, structural characterization, etc.), e.g., by considering the charge of the nickel reduction wave as a measure of the electrochemically active amount. After activation, the electrocatalyst films were cyclovoltammetrically characterized at scan rates of 10 mV s^–1^ over 50 cycles for their OER-catalytic activity, evaluating, e.g., Tafel slopes. For ex situ structural investigations, nickel selenide films were electrodeposited on gold-coated glass slides, and characterization measurements were carried out on the pristine catalyst films after testing the alkaline OER performance. As can be seen from the SEM images in Figure 2a, the electrodeposition process yields films of granular particles in the size range of tens to hundreds of nanometers. The morphology of the catalyst films did not change after the electrochemical measurements.

**Figure 2:**
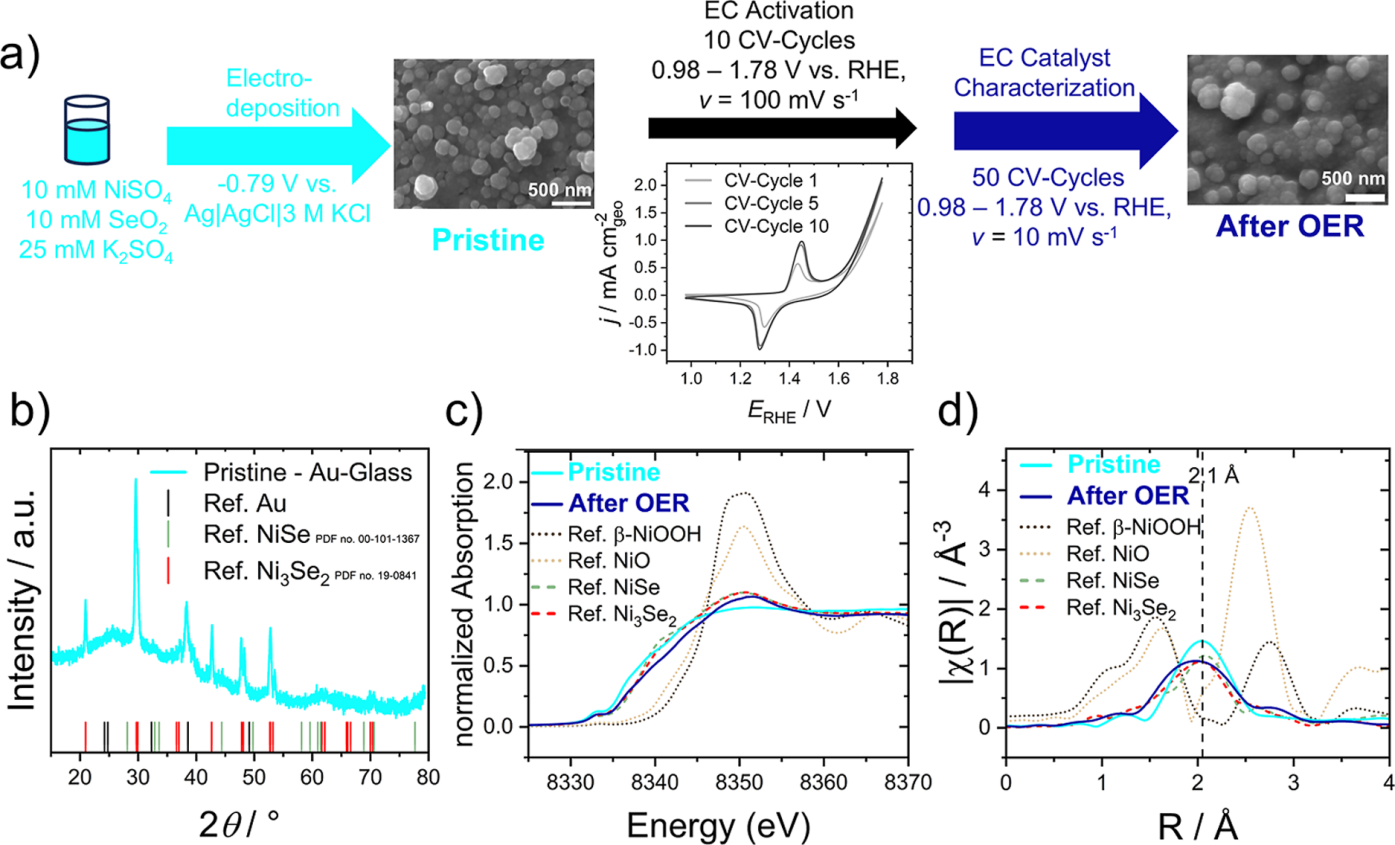
Morphological and structural characterization of nickel selenide films after electrodeposition (pristine) and after evaluation of the OER activity (after OER). (a) Schematic illustration of the experimental deposition and OER procedure including SEM images of the films before and after full electrochemical characterization; (b) X-ray diffraction (XRD) pattern of the pristine catalyst film displaying known diffraction peaks of NiSe (PDF no. 00-101-1367), Ni_3_Se_2_ (PDF no. 19-0841), and Au^40^ (from the substrate); (c) X-ray absorption near structure (XANES) region of XAS spectra at the Ni K-edge (8325—ca. 8370 eV), (d) Fourier transforms of Ni K-edge extended X-ray absorption fine structure (EXAFS) spectra in *R*-space.

The observed peaks in the XRD pattern of the pristine catalyst film in Figure 2b match the reference pattern for rhombohedral Ni_3_Se_2_ (PDF no. 19-0841), while signals from the Au substrate were distinguishable. No additional crystalline phases were detected. Since XRD is a bulk-sensitive technique, we conclude that Ni_3_Se_2_ is dominant in the bulk structure of the deposit, in agreement with observations by Cao et al.^8^

XAS has been widely utilized to study the evolution of the chemical state of (pre)­catalyst materials during their transformation under catalytic processes.^41,42^ Figure 2c,d shows the normalized XANES and Fourier-transformed EXAFS data in *R*-space, respectively, of the pristine electrodeposited catalyst and after the alkaline OER test. The spectra for the sample in the pristine state and after the OER agree with those from NiSe and Ni_3_Se_2_ reference nanoparticles (see Supporting Information-Section 1.5.3) in the XANES regions, indicating a similar phase and composition. In addition, the pre-edge features of both pristine and after OER spectra at ≈8333 eV resemble those of NiSe and Ni_3_Se_2_ references, suggesting a similar local site symmetry and coordination. Based on the assumed deposition mechanism, Ni_3_Se_2_ is formed from NiSe.^43^ The Ni K-edge in EXAFS *R*-space indicates a single strong signal at ≈2.1 Å for both pristine catalyst and after OER, which can be assigned to the Ni–Se bond distance in nickel selenide materials.^44,45^ The Ni K-edge in *k*-space spectra (Figure S10b) is also in good agreement with the NiSe and Ni_3_Se_2_ reference samples.

In summary, the XAS measurements support the electrodeposition of a nickel selenide species. Since the XAS spectrum resembles a fingerprint of the specific chemical environment for each absorbing atom, we can investigate the proportions of chemically distinct Ni sites in our samples using a linear combination fitting analysis, as described in Supporting Information-Section 1.5.4. The XANES spectrum of the after OER sample reveals the presence of a nickel oxide or oxyhydroxide, with an estimated composition of ≈20% NiO and ≈80% of pristine nickel selenide species (Figure S10a). In contrast, the pristine films showed no oxide features.

XPS measurements were conducted for the Ni 2p_3/2_ and Se 3d signals to determine the surface content of nickel and selenium in pristine and electrochemically characterized films (Figure 3a–d). Assignments of the different nickel selenide species were performed using the Se 3d signal as described in the Electronic Supporting Information where additional information on the XPS data processing and analyses can be found (Supporting Information-Section 1.5.5).^46,47^

**Figure 3:**
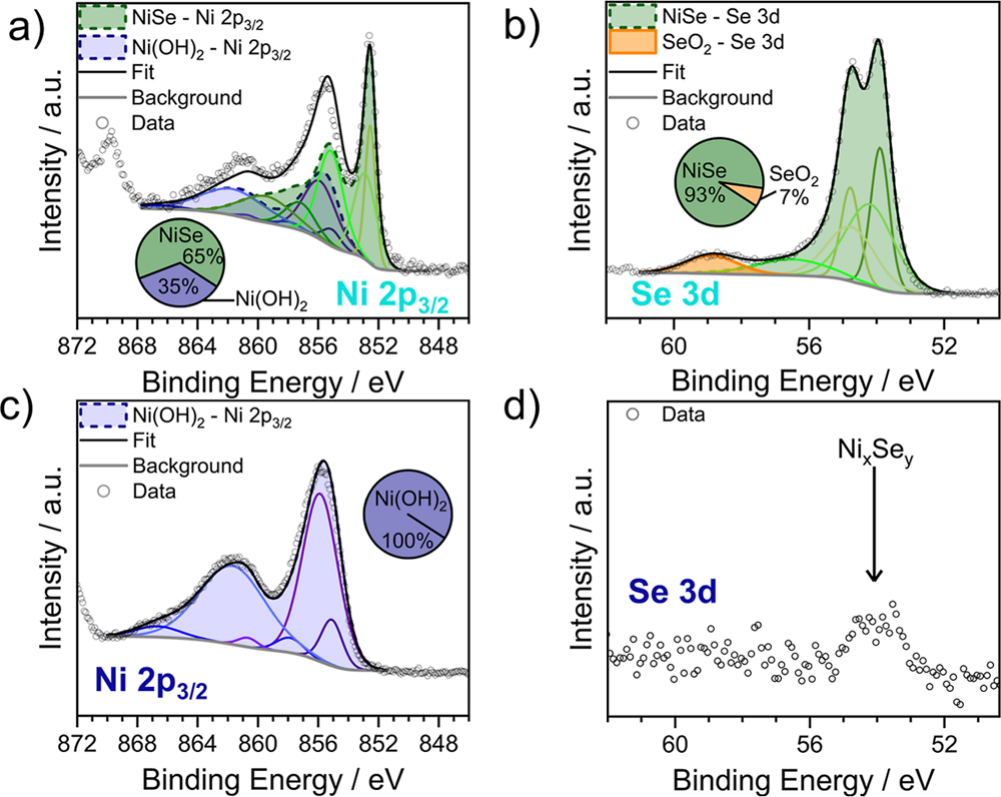
XPS spectra of (a,c) the Ni 2p_3/2_ and (b,d) the Se 3d signals of (a,b) the pristine catalyst film and (c,d) the film after OER testing.

The Ni 2p_3/2_ signal in the pristine material (Figure 3a) reveals the presence of two chemical states, namely, NiSe (≈65%) and Ni­(OH)_2_ (≈35%). Notably, XPS is a surface-sensitive technique^46,48^ which explains why hydroxide signals were already found in the pristine sample, whereas no oxide species were detected in the bulk-sensitive XAS characterization^49^ of the pristine deposit. Although distinguishing between Ni­(OH)_2_ and NiOOH in XPS experiments is not straightforward^50^ and alternative nickel oxide species cannot be safely excluded by the analyses of the present data (see Supporting Information-Section 1.5.5.1),^46^ the predominant appearance of Ni­(OH)_2_ under ex situ conditions seems reasonable since NiOOH samples tend to self-discharge to Ni­(OH)_2_ with time.^51^ The dominance of NiSe at the catalyst surface and Ni_3_Se_2_ as the bulk structure (XRD pattern Figure 2b) is consistent with previous reports that Ni_3_Se_2_ is formed from NiSe during electrodeposition.^43^ To prove this assumption, we conducted Ar-sputtering of the pristine catalyst surface to remove the uppermost layers (see Supporting Information-Section 1.5.5 for details). In the surface underlying layers, we found both materials, NiSe and Ni_3_Se_2_ (Figure S5b).

The Se 3d signal of the pristine catalyst (Figure 3b) displays a pronounced doublet related to Se 3 d_5/2_ and Se 3 d_3/2_ in nickel selenides^52^ as well as a weaker doublet which we assigned to selenide oxides.^53^ Due to the weak Se 3d signal in the spectra of the nickel selenide film after the OER (Figure 3c), the Se 3d peak was not fitted as the signal-to-noise ratio was deemed insufficient for meaningful analysis. However, the position of the peak at approximately 54 eV suggests that the signal originates from a nickel selenide species. Concurrently, the oxygen content in the after OER samples increased (Supporting Information Table 12), and the Ni 2p_3/2_ signal (Figure 3d) indicates an increase in Ni­(OH)_2_ (see Supporting Information-Section 1.5.5 for details), consistent with the recognized precatalyst transformations for nickel selenides.^5,7,9,11^

In summary, the combined XRD, XPS, and XAS results consistently demonstrate a surface-bulk phase gradient typical for electrodeposited nickel selenides. The pristine electrodeposited nickel selenide films consist of spherical nano- and submicrometer particles of Ni_3_Se_2_ in the bulk and NiSe and Ni­(OH)_2_ on their surface. When the OER-electrocatalytic properties are studied in an alkaline electrolyte, this precatalyst material is partially oxidized to nickel oxide and/or hydroxide species, while selenium is partially leached out and the morphology is retained. Next, the electrocatalytic activity toward the OER and its development have been characterized by CV under conditions of high mass transport, and thus, high current densities, using microelectrodes.

### 3.2 Electrochemical Characterization via Cyclic Voltammetry

As can be seen in Figure 4a, the overpotential and Tafel slope of the electrodeposited and activated nickel selenides (see Figure S12 for the CV activation period) are enhanced, especially during the first part of the 50 CV cycles. This indicates that the activation and hence the conversion of the nickel selenides continue to progress. Surprisingly, at more positive (anodic) potentials, the (mass-transport-limited) current density decreases throughout the cycling.

**Figure 4:**
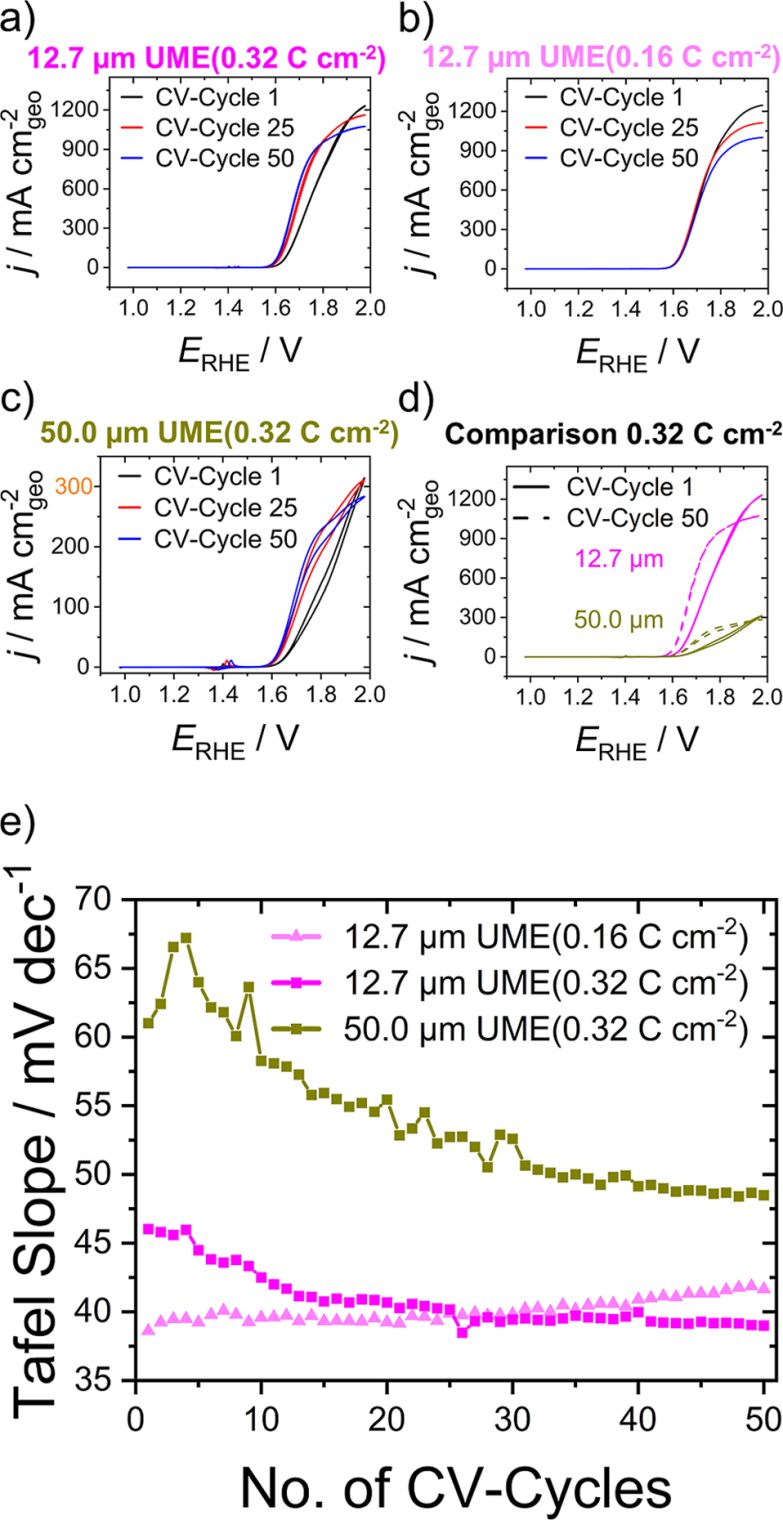
Electrochemical characterization of nickel selenides under OER conditions at 10 mV s^–1^ in 0.1 M KOH with 1 mM Fe­(NO_3_)_3_. (a,c,d) CV curves for high catalyst loading (0.32 C cm^–2^) on (a) 12.7 μm and (c) 50.0 μm UMEs, whereas (d) shows the comparison of the first and 50th CV cycle; (b) CV curves for low catalyst loading (0.16 C cm^–2^) on a 12.7 μm UME; (e) change of Tafel slopes extracted from CV curves over successive cycles from experiments in (a–c).

When half of the catalyst loading is chosen (Figure 4b), the kinetically controlled region is rather unaffected, while the steady-state current decreases even more significantly. At 1.98 V vs RHE, the current density decreases by approximately 0.2 A cm^–2^ from the first to the 50th CV cycle.

This nontypical behavior was investigated further using a larger microelectrode (Figure 4c,d, 50.0 μm electrode diameter) to observe the effect at high (> 100 mA cm^–2^) but significantly lower current densities than 1 A cm^–2^. This was possible as the mass-transport-limited steady-state current of UMEs scales with the electrode diameter. As expected, the current density reached at 1.98 V vs RHE decreases from 1.2 A cm^–2^ for the smaller electrode (12.7 μm) to 0.3 A cm^–2^ for the larger electrode (50.0 μm). During cycling, the CVs conducted with the larger microelectrode also become sigmoidally shaped, and the current density determined by mass transport decreases. Again, the overpotential and Tafel slope decrease (Figure 4e). Furthermore, a shift of the nickel oxidation wave to more positive potentials is observed, which has been attributed to increasing iron incorporation in nickel oxide/oxyhydroxide-based catalyst films in previous studies.^16,17,54^

These findings show that further conversion of the nickel selenides to the electrocatalytically active species is affected by mass transport. For the low catalyst loading, the conversion was almost complete after the initial activation and did not continue much during the OER-activity assessment. Conversely, further activation was observed at a higher catalyst loading. Comparison of fast mass transport conditions (12.7 μm electrode) with intermediate mass transport (50.0 μm electrode, Figure 4d,e) then suggests a difference in catalyst activation, although it is generally observed for both. Summarized, the development of Tafel slopes with ongoing CV cycling shows that the kinetics of the catalyst system improve within the first ca. 25 cycles and are constant in the following 25 for the high loadings. Thus, we conclude that the decrease in the current density at high overpotentials for the small (12.7 μm) UME with high loading does not originate from kinetic degradation.

However, the consistent “degradation” of the mass-transport-limited current density with CV cycle number is very surprising. A change in the electrode diameter of the microelectrodes seems to be very unlikely. We therefore investigate further the reason for this observation.

### 3.3 Electrochemical Characterization via Electrochemical Impedance Spectroscopy

EIS measurements were performed under OER conditions at 1.8 V after the electrochemical activation period, after 25 cycles, and after 50 cycles of the CV characterization experiments. As shown in Figure 5 (see Figures S14 and S15 for data for the lower loading of 0.16 C cm^–2^), at least two semicircles can always be distinguished in the complex plane plot.

**Figure 5:**
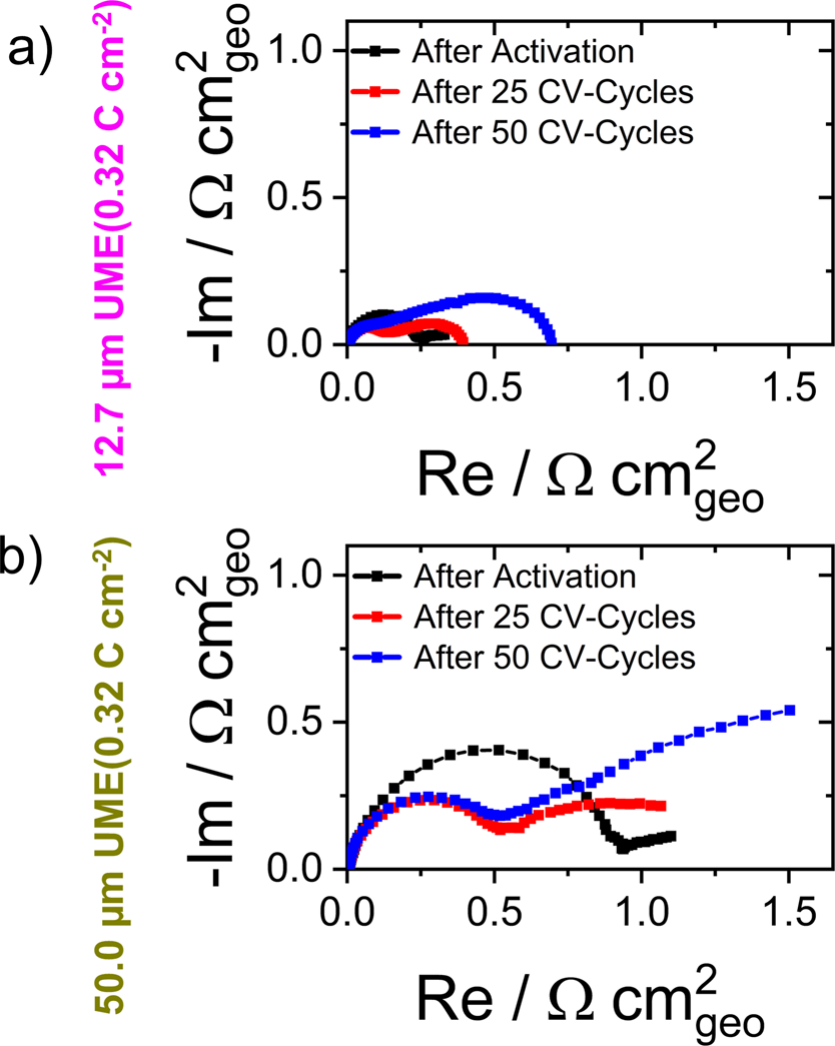
Nyquist plots of EIS spectra recorded at 1.8 V vs RHE after activation, after 25 CV cycles and 50 CV cycles of OER characterization for a deposited catalyst loading of 0.32 C cm^–2^ on (a) 12.7 μm and (b) 50.0 μm UMEs.

The semicircle appearing at high frequencies is attributed to the charge transfer processes of the electrocatalytic reaction in parallel with the capacitive charging.^55,56^ Accordingly, in Figure 5, its diameter, corresponding to the charge-transfer resistance, decreases for the first cycles as further activation toward OER electrocatalysis was observed in the CV measurements. Furthermore, the semicircle diameters in the high frequency part are larger for the 50 μm electrode than those for the 12.7 μm electrode, in agreement with the voltammetry results (Figure 4d,e).

The lower-frequency parts of the spectra are assumed to be related to the mass transport behavior.^55,56^ For convergent diffusion, a distorted semicircle is expected, with the diameter of the semicircle corresponding to the diffusion resistance.^57^ The trends observed with increasing numbers of cycles in Figure 5 are consistent with this assignment as increasing diffusion resistances are observed where mass-transport-limited currents decrease in the CVs in Figure 4.

In addition, the semicircle diameters for the lower frequencies are smaller for the 12.7 μm electrode than that for the 50 μm electrode, where the measured frequency range was too limited to observe “closed” semicircles intersecting the real axis. This agrees with the faster mass transport at smaller microelectrodes.^55,57–59^

To identify the reason for the “mass transport degradation” under the conditions of high electrocatalytic current densities, we look in detail at the impedance spectra recorded after 50 cycles (shown for different axis scales in Figure 6a,b). For clarity, the frequency ranges we refer to are shown in different colors (high—red, moderate—orange, and low—green).

**Figure 6:**
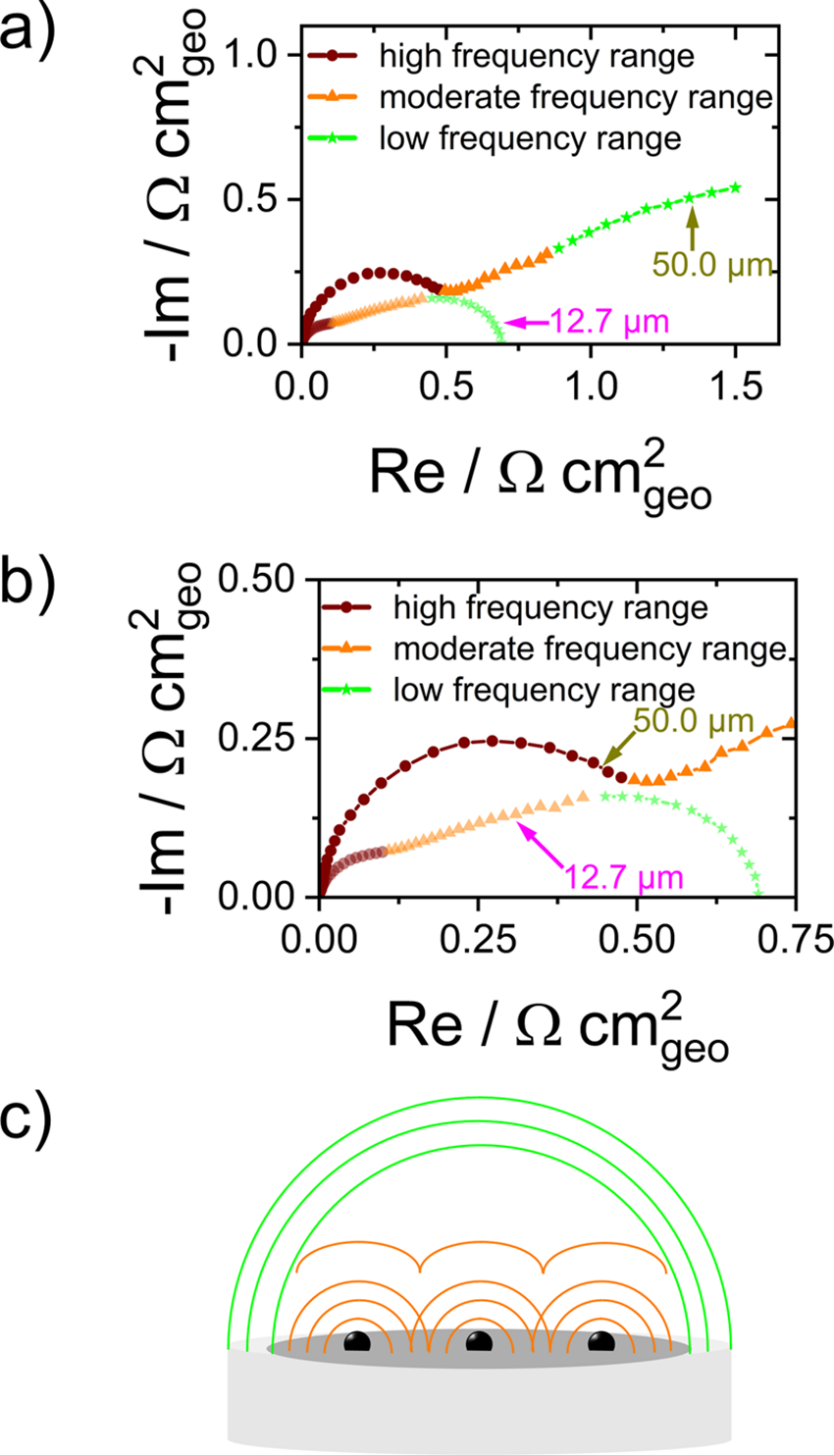
(a) Nyquist plots of EIS spectra after 50 CV cycles of OER characterization for an electrodeposited catalyst loading of 0.32 C cm^–2^ on 12.7 μm (transparent colors) and 50.0 μm (solid colors) UMEs with high-, moderate-, and low-frequency regions highlighted; (b) magnified view of the EIS spectra from (a); (c) schematic illustration of an UME, depicting mass transport profiles on granular nanoparticles and across the entire UME.

The part of the EIS spectra assigned to mass transport (here moderate and low frequencies) shows two distinct sections for the progressed degradation in terms of reduced mass-transport-controlled current. Due to the nanoparticulate morphology of the electrodeposited nickel selenides, we do not only consider convergent diffusion to the microelectrodes but also to the nanoparticles,^30,60–62^ and thus a different scale and coupled diffusion phenomena (Figure 6c). It is assumed that diffusion to nanoparticles at this scale is initially so fast that the corresponding resistances cannot be identified in the EIS spectra. However, after 50 cycles, the morphology may have changed in terms of particle sizes, spacing between particles, or disappearing spherical shapes.

To confirm this hypothesis, we carried out equivalent circuit fitting with a simple model (Figure S16), where convergent diffusion phenomena are represented by parallel constant phase element resistor circuits, which are in series with the electrolyte and charge transfer resistors. As shown in Table S13, the series resistances, charge transfer resistances, and diffusion resistances to the microelectrodes show the expected differences for the two microelectrode sizes.

The geometric-area-normalized diffusion resistance to the microelectrode *R*_D,UME_ is 1 order of magnitude larger for the 50 μm electrode than that for the 12.7 μm electrode. For the smaller microelectrode (12.7 μm diameter), which shows the most pronounced degradation effect, the diffusion resistance to catalyst particles *R*_D,NP_ is in the same order of magnitude as the diffusion resistance to microelectrode *R*_D,UME_. The higher diffusion resistance toward single nanoparticles *R*_D,NP_ relative to the total microelectrode *R*_D,UME_ for the smaller UME (12.7 μm) suggests that the diffusion profiles of individual nanoparticles overlap to a continuous layer, which would hamper the mass flux and thus current flow at high overpotentials. The origin of the merging of the diffusion layers could be an alteration of the nanoparticle’s morphology to larger structures, e.g., by aggregation.^63,64^ Therefore, we conclude that a change in the catalyst film morphology causes the observed decreased mass flow as a degradation effect at high catalytic current densities.^65^ To verify this assumption, SEM measurements were taken at the same location on the microelectrodes.

### 3.4 Morphological and Compositional Analyses via Identical Location Scanning Electron Microscopy/Energy-Dispersive X-ray Spectroscopy

Figure 7 shows SEM images of identical locations on a 12.7 μm electrode and Figure 8 on a 50 μm electrode before and after the characterization of the electrocatalytic activity toward the OER. The full SEM overview images (Figure S17) and atomic shares extracted from the respective EDX spectra (Table S14) are provided in the Supporting Information.

**Figure 7:**
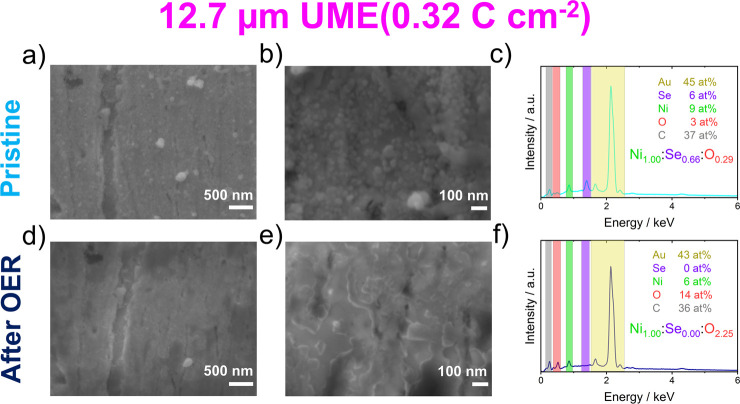
SEM images and EDX spectra of a 12.7 μm UME of the (high loading, 0.32 C cm^–2^) (a,c,e) pristine-deposited film and (b,d,f) the same film after OER testing.

**Figure 8:**
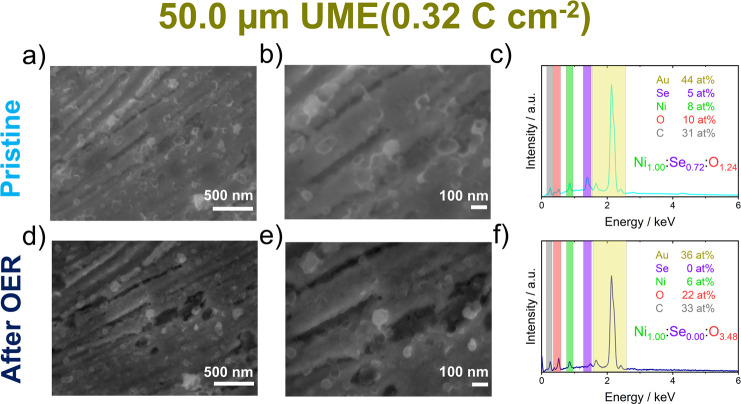
SEM images and EDX spectra of a 50.0 μm UME of the (high loading, 0.32 C cm^–2^) (a,c,e) pristine-deposited film and the (b,d,f) same film after OER testing.

For the smaller microelectrode, where current densities higher than 1 A cm^–2^ were recorded, a drastic change in the catalyst film morphology from the sample in the pristine state (Figure 7a,b) to after the OER state (Figure 7d,e) is observed. Initially, spherical and ellipsoidal particles of sizes well below 100 nm to a few hundred nanometers are seen. Afterward, electrocatalytic testing (Supporting Information-Section 1.4) of this UME was performed to allow the imaging of the same spot using (identical location) SEM and to investigate potential morphological changes related to the material transformation under OER conditions. The sample after OER exhibits only flat and broad patches, whereas nearly all spherically shaped particles have disappeared.

On the larger microelectrode (50 μm diameter) providing for an order-of-magnitude lower current density, particle morphology is still clearly visible after assessment of the electrocatalytic activity (Figure 8).

In addition to SEM imaging, the composition of the films on the microelectrodes was investigated by EDX (Figures 7c,f and 8c,f). A Ni/Se atomic ratio of ≈3:2 was found for both microelectrode sizes, again supporting the idea that Ni_3_Se_2_ is the dominant bulk species in the electrodeposited film (see XRD in Figure 2b). The Se content diminished after electrocatalytic characterization accompanied by a significant increase in the oxygen content, confirming the transformation into nickel oxide/oxyhydroxide.^5,7,9,11^ Further, this indicates that the share of transformed nickel selenide material in our UME experiments is higher than that on a macro electrode substrate (Section 3.1). Noteworthy, the spectroscopic analyses of the after OER samples on macro electrode substrates (Figures 2 and 3) show a significant share of untransformed nickel selenide alongside nickel oxide/oxyhydroxide species. These deviations can be explained by the thinner film loading and improved mass transport conditions (convergent diffusion) in the case of the UME experiments.

A significant loss of catalyst material by oxygen bubble formation seems unlikely since the absolute current flowing at the small UME (12.7 μm) is small (<1.5 μA) and the mass flux is enhanced due to the convergent diffusion profile. Thus, the formation of oxygen bubbles—which is commonly observed in high amounts on macro electrodes for gas-evolving reactions—is rare on UMEs.^66^

To elaborate more details on the degradation mechanism of nickel selenide-based OER electrocatalysts at current densities of industrial relevance, experiments with coupled electrochemical and microscopic techniques to follow the catalyst composition and morphology, e.g., in situ TEM,^65^ are needed. A potentially attractive electrochemical method might be scanning electrochemical cell microscopy (SECCM). In this technique, a pipet with a diameter down to tens of nanometers allows the electrochemical monitoring of individual (nano-) particles on a support surface and their changes under applied potential.^67^ However, the stability of the electrolyte droplet is a significant problem for SECCM investigations, especially in alkaline media where the addition of organic additives to the electrolyte is required.^68^

## 4 Conclusion

We successfully used the unique environment of UMEs, enabling electrochemical investigations of materials at current densities of industrial interest, accompanied by a negligible influence of the ohmic drop, to study electrodeposited nickel selenide films under alkaline OER conditions. UMEs of two different diameters (12.7 and 50.0 μm) were used to study activation, originating from the transformation to oxide/oxyhydroxide species during anodic potential sweeping and degradation of electrodeposited nickel selenide nanoparticle films. Due to the smaller diameter, higher current densities were achieved in these CV studies on the 12.7 μm compared to the 50.0 μm UME. For both UMEs, charge transfer kinetics improved with increasing reaction times due to successive precatalyst transformation to the OER active species. Notably, for current densities exceeding 1 A cm^–2^, the current density at high OER overpotentials dropped with increasing experimental times due to hitherto unrecognized morphological changes of the catalyst. Using EIS measurements, we identified that this originates from the merging of diffusion zones at individual nanocatalyst particles caused by an increase in the particle size with increasing experimental times.

This was confirmed by identical location SEM, which showed severe morphological changes of the nickel-selenide-based catalyst material at such high current densities. Morphologic degradation has been reported for other catalyst systems, e.g., for Co_3_O_4_ nanoparticles, which also transform into an oxide/oxyhydroxide under OER conditions.^65^ Conversely, for nanoparticulate nickel selenide catalysts, severe morphology alterations have not been reported before, presumably caused by the fact that they only occur at current densities exceeding those of most previous studies.

Since most studies on chalcogen-based OER electrocatalysts are conducted at current densities far below industrial relevance, the occurrence of morphological catalyst degradation under application-relevant conditions might have been vastly underestimated. Having demonstrated the severe effect of morphological changes for nickel-selenide-based OER catalysts in this work, we suspect that this might similarly apply to other transition-metal chalcogenides and should be considered during the development of improved catalyst materials for alkaline electrolyzers.

## Supplementary Material









## Notes

Detailed experimental section with the full electrochemical protocol, additional electrochemical activity data, SEM, EDX data, and a brief description of the equivalent circuit and fitting data of EIS analysis (PDF)
Crystallographic data of Au (CIF)
Crystallographic data of NiSe (CIF)
Crystallographic data of Ni_3_Se_2_ (CIF)

## ⊥

Faculty of Mechanical Engineering, Chair of Atomic Scale Characterization, Surface Science & Electrochemistry, Ruhr University Bochum, Bochum, 44801, Germany

## Abbreviations

OER: oxygen evolution reaction
UME: ultramicroelectrode
RDE: rotating disk electrode
EIS: electrochemical impedance spectroscopy
XAS: X-ray absorption spectroscopy
XPS: X-ray photoelectron spectroscopy
XRD: X-ray diffraction
SEM: scanning electron microscopy
EDX: energy-dispersive X-ray spectroscopy
GDE: gas diffusion electrode
MEA: membrane electrode assemblies
TEM: transmission electron microscopy
SECCM: scanning electrochemical cell microscopy
