# Supplementary material for: Morphological Degradation of Oxygen Evolution Reaction-Electrocatalyzing Nickel Selenides at Industrially Relevant Current Densities
Source: ACS Appl Mater Interfaces. 2025 Jul 11;17(29):41893–903. doi: 10.1021/acsami.5c05381 (PMC12291084; doi:10.1021/acsami.5c05381)
Supplement: Supplementary file 1 [file am5c05381_si_001.pdf]

Supporting Information (SI):

Morphological Degradation of OER-Electrocatalyzing Nickel Selenides at Industrially Relevant Current Densities

Felix Hiege,[a] Chun-Wai Chang,[b] Oliver Trost,[a] Charlotte E. R. van Halteren,[c] Pouya Hosseini,[d,†] Georg Bendt,[c] Stephan Schulz,[c] Zhenxing Feng[b] Julia Linnemann,[a]* and Kristina Tschulik [a,d]*

[a]Faculty of Chemistry and Biochemistry, Chair of Analytical Chemistry II, Ruhr University Bochum, Bochum, North Rhine-Westphalia, 44801, Germany. *corresponding author: E-mail: julia.linnemann@rub.de;

kristina.tschulik@rub.de

[b]School of Chemical, Biological, and Environmental Engineering, Oregon State University, Corvallis, Oregon, 97331, USA.

[c]Faculty of Chemistry, Chair of Inorganic Chemistry, and Center for Nanointegration Duisburg-Essen (Cenide), University Duisburg-Essen, Essen, North Rhine-Westphalia, 45141, Germany.

[d]Max-Planck-Institut für Nachhaltige Materialien GmbH, Max-Planck-Straße 1, Düsseldorf, North Rhine-Westphalia, 40237, Germany

1.1 Electrochemical Setup

All electrochemical measurements were conducted with a *ModuLab XM ECS Solartron Analytical potentiostat* (Model: XM CHAS 08) by *AMETEK Scientific Instruments*. The high voltage/ high impedance / low current connection of the *ModuLab XM ECS Solartron Analytical* was used at a bandwidth of 100 kHz in a grounded Faraday cage. A Pt wire (d = 1 mm, *Alfa Aesar*; 99.95 % metal-based) and a homemade Ag|AgCl|3 M KCl were used as counter (CE) and reference electrode (RE). To ensure an accurate reference potential, the potential of RE was checked before and after any experiment and a double junction was used to avoid contamination of the electrolyte. For the electrochemical impedance measurements of the OER in alkaline media a Pt wire (d = 0.25 mm, *Alfa Aesar*; 99.99 % metal-based) and a 100-pF bypass capacitor were added to the RE to suppress the influence of stray capacitances at high frequencies. All electrochemical measurements were performed in an alkali-resistant polymer cell (Eppendorf).

1.2 Fabrication and Pre-Treatment of Ultramicroelectrodes (UMEs):

For the fabrication of the UMEs, gold wires with a diameter of 12.7 µm and 50 µm (*Alfa Aesar*; 99.99 % metal-based) were used. First, one end of the wire was inserted into a soda-lime glass capillary (*Hilgenberg*; d_{outer} = 1.0 mm, d_{inner} = 0.25 mm) using vacuum pump suction until the end of the capillary was closed. Afterward, the 'loaded' soda-lime glass capillary was inserted in the middle of a heating coil (R = 0.293 Ω) and heated under vacuum (pressure $< 3.0\cdot10^{-2}$ mbar) to seal the capillary around the gold wire at 14 A heating current for 2 min. A silver wire (d = 0.25 mm, *Thermo Scientific Chemicals*, 99.9 % metal basis) was inserted inside the open end of the capillary for the electrical connection to the potentiostat. It was further

annealed under vacuum at 17 A for 1 minute at the contact spot of both wires (gold and silver) to ensure good electrical connection.

The such fabricated UMEs were polished using sandpapers with decreasing roughness (from P600 to P7000; Starcke) and afterward polished with 1.0 µm and 0.3 µm Al_2O_3 lapping paper (3M) for 3 minutes each. To remove residuals of Al_2O_3 lapping paper, the UMEs were sonicated (*Elamsonic S 100 H*) for 20 seconds in ultra-pure water (coductivity< 0.056 µS cm^{-2} at 25°C, Thermo Scientific). Additionally, the UMEs were dipped in freshly prepared aqua regia (H_2SO_4 : H_2O_2 3:1) shortly before electrochemical measurements to remove contaminations and cleaned in water before use.

Before the electrodeposition of nickel selenides, 20 CV cycles in a potential window of −0.1 to 1.45 V vs. Ag|AgCl|3 M KCl (start/end potential 0.2 V) at a scan rate of 100 mV s^{-1} in aq. 0.5 M H_2SO_4 on UMEs were monitored to 1) achieve a clean working electrode by multiple oxidation and reduction of the gold surface and 2) estimate the microscopic area by integrating the gold reduction peak of the last CV cycle and dividing it by the theoretically estimated specific charge of a chemisorbed gold oxide monolayer on a polycrystalline gold surface of 482 µC cm^{-2}.[1] The experimental cleaning CVs and the calculated microscopic areas Am of the UMEs used in this study are provided in Figure S1.

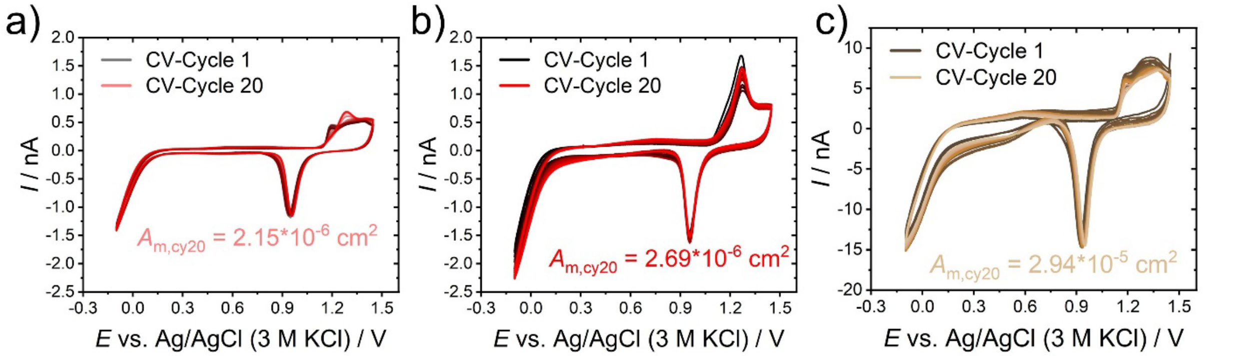

Figure S1. Electrochemical cleaning prior to electrodeposition of the a) 12.7 µm (0.16 C cm^{-2} catalyst deposited afterward), b) 12.7 µm (0.32 C cm^{-2} catalyst deposited afterward) and c) 50.0 µm (0.32 C cm^{-2} catalyst deposited afterward) UMEs. Each electrode was treated in a 20 CV-cycles-measurement in a potential window of −0.1 to 1.45 V vs. Ag|AgCl|3 M KCl (start/end potential 0.2 V) at a scan rate of 100 mV s^{-1} in 0.5 M H_2SO_4 on every UME were monitored to 1) clean its surface by multiple oxidation and reduction of the gold surface and 2) estimate the microscopic area Am (given in each Figure a), b) and c)) by integrating the gold reduction peak of the last CV cycle.

1.3 Electrodeposition of Nickel Selenides

The electrodeposition procedure of nickel selenides was selected based on the procedure of Cao et al.[2] The precursor solutions were 150 mM $NiSO_4 \cdot 6H_2O$ (*Sigma Aldrich*, ≥99.99 % trace metal basis), 150 mM SeO_2 (*Sigma Aldrich*, 99.9 % metal basis), and 375 mM K_2SO_4 (*Supleco*) in ultra-pure water (< 0.056 µS cm at 25 °C). For the electrodeposition of nickel selenide, 1 mL of each stock solution was mixed with 12 mL of ultra-pure water to get a 15 mL solution with concentrations of 10 mM $NiSO_4$, 10 mM SeO_2, and 25 mM K_2SO_4 (pH 2.5). Afterward, the solution was deoxygenated under Ar-flow for at least 20 minutes. For every electrodeposition experiment, 2 mL of the deoxygenated solution was used.

The electrodepositions were performed in chronoamperometric experiments using the three-electrode setup as described above at −0.79 V vs. Ag|AgCl|3 M KCl with coulometric termination control. Additionally, a double junction was linked to the RE, to prevent chloride contamination of the KOH solution.

The coulometric charge loadings selected in the conducted experiments were 158 mC cm^{-2} (only for UME with d = 12.7 µm, and 316 mC cm^{-2} (for both UME sizes, d = 12.7 and d = 50 µm) calculated based on the geometrical sizes of the UMEs.

1.4 Electrochemical Investigation of OER-Electrocatalytic Activity, Activation and Degradation in Alkaline Electrolytes

The electrocatalytic activity of the electrodeposited (pre-)catalysts towards OER was investigated in aq. 0.1 M KOH (99.99 % trace metal basis, *Sigma Aldrich*) with the addition of 1 mM $Fe(NO_3)_3$ (98 % ACS reagent grade, *Sigma Aldrich*). 24 h after initial preparation of the electrolyte, the $Fe(OH)_3$ precipitants were removed.[3] The concentration of ferric ions in the solution was determined as ≈ 14 µmol L^{-1} using inductively coupled plasma mass spectrometry (ICP-MS). Reproducibility is enhanced with this approach of an intentionally increased iron concentration in the electrolyte as unpredictable incorporation of iron contaminations is avoided.

The automated electrochemical measurement protocol consisted of 14 steps (where OCP means measuring the open circuit potential, EIS electrochemical impedance spectroscopy, and CV cyclic voltammetry):

1. OCP, 2 min
2. EIS @ OCP

3. 10 CV cycles @ 10 mV s^{-1}, 0 – 1.0 V vs. Ag|AgCl|3 M KCl

4. OCP, 5 min

5. EIS @ 1.8 V vs. RHE (0.82 V vs. Ag|AgCl|3 M KCl)

6. OCP, 5 min

7. 25 CV cycles @ 10 mV s^{-1}, 0 – 1.0 V vs. Ag|AgCl|3 M KCl

8. OCP, 5 min

9. EIS @ 1.8 V vs. RHE (0.82 V vs. Ag|AgCl|3 M KCl)

10. OCP, 5 min

11. 25 CV cycles @ 10 mV s^{-1}, 0 – 1.0 V vs. Ag|AgCl|3 M KCl

12. OCP, 5 min

13. EIS @ 1.8 V vs. RHE (0.82 V vs. Ag|AgCl|3 M KCl)

14. OCP, 5 min

The EIS measurements were conducted with an amplitude of 7.1 mV (rms) in a frequency range from 100 kHz to 0.1 Hz in the frequency sweep mode of the *ModuLab XM ECS Solartron Analytical*.

For displaying electrochemical data, electrochemical potentials were converted from Ag|AgCl|3 M KCl to RHE scale[4] using the equation (S1):

$$E_{RHE} = E_{(Ag|AgCl|3MKCl)} + (0.059 \cdot pH) + 0.207\ \mathrm{V} \tag{S1}$$

Tafel slopes were extracted using a Python code provided to us by Dr. Kannasoot Kanokanchana (TU München, Germany), which estimated the Tafel slopes by the optimization of the linear fit R^2 of Tafel plots in the potential range of 50 mV between 1.45 and 1.71 V vs. RHE. At these relatively low potentials, charge-transfer kinetics were assumed to be rate-limiting. Analysis of impedance data was performed using *RelaxIS 3* (*rhd instruments*). For data processing, a Z-HIT algorithm was used. For further data analysis and construction of figures, *OriginPro 2021b* was used.

1.5 Morphological, Structural, and Compositional Characterization of Electrodeposited Nickel Selenide Electrocatalysts

The electrochemical deposition procedure (SI-Section 1.3) was performed on gold macroelectrodes to subject these specimens to characterization measurements for all morphological, structural, and compositional characterization of electrodeposited nickel selenide films. Exceptions are the identical location SEM (IL-SEM) investigations on the two UMEs (SI-Section 1.5.1). Gold-coated glass plates (A = 0.283 cm^2) were fabricated by thermal evaporation (≈ 200 nm gold on ≈ 8 nm titanium, adhesion layer). Before electrodeposition, the plates were cleaned through sonication in isopropanol (ACS grade; Sigma Aldrich) for 3 minutes. Next, electrodeposition was performed in a homemade Teflon cell. The Au-coated glass plates were fixed at the bottom of the cell. For the electrodeposition on the macroelectrode gold-coated glass substrates, similar CE and RE as well as deposition potential were used as for the deposition on UME, but the deposition time was extended to 600 s to completely cover the substrate with a thick and dense catalyst layer.

1.5.1 SEM/EDX Measurements

Scanning electron microscopy (SEM) investigations on UMEs were performed using a high-resolution scanning electron microscope *JSM-IT800* (*JEOL*) at 10 kV acceleration voltage. A homemade holder consisting of stainless steel was used to image the surface of the UME at a tilt angle of 0°.

For identical location experiments, the same UME was investigated first after mechanical polishing, second after electrodeposition of nickel selenides, and third after electrochemical characterization in alkaline electrolyte.

An *Oxford Ultim Max Silicon Drift Detector* (SDD) at 10 kV was used as primary detector to monitor energy dispersive X-ray (EDX) spectra. Data processing and analysis of the EDX spectra was performed using *Oxford AZtecLive*.

1.5.2 XRD Measurements

Crystallographic phase analysis was conducted using a Bruker D8 Discover diffractometer (Bruker Corporation) in Bragg–Brentano geometry, equipped with a VANTEC-500 area detector and a Cu Kα radiation source (λ = 1.5406 Å) operated at 36 kV and 36 mA. The sample-to-detector distance was set to 149 mm. The incident X-ray beam was collimated to a diameter of 1 mm with a divergence angle below 0.007°, ensuring high angular resolution. Diffraction data were collected in three individual frames per sample position, the collected frames were integrated into 1D diffractograms using DIFFRAC.EVA software (Bruker Corporation) for subsequent phase identification and analysis. The angular accuracy of the instrument was within ±0.02° 2θ. To avoid Au-substrate peaks, measurements were performed in θ–2θ mode. There the

X-ray source is fixed in position, while the sample rotates with θ °/min and the 2D-detector with 2θ °/min.

1.5.3 Synthesis and XRD Characterization of NiSe and Ni_3Se_2 Reference Samples

Reference samples of NiSe (hexagonal phase, phase pure), and Ni_3Se_2 (rhombohedral phase (main) with hexagonal NiSe phase (minor), were synthesized via a solvothermal approach following Yuan et al.[5]. The reference samples were characterized using powder XRD (PXRD) conducted on glass or silicon objectives. The corresponding patterns of NiSe and Ni_3Se_2 are shown in Figure S2. The crystallographic CIF files of NiSe ("NiSe_PDF_00-101-1367_COD_9008904.cif") and Ni_3Se_2 ("Ni3Se2_PDF_19-0841_COD_9009243.cif") used to assign the signals of the experimental XRD pattern of the synthesized reference materials, are additionally provided.

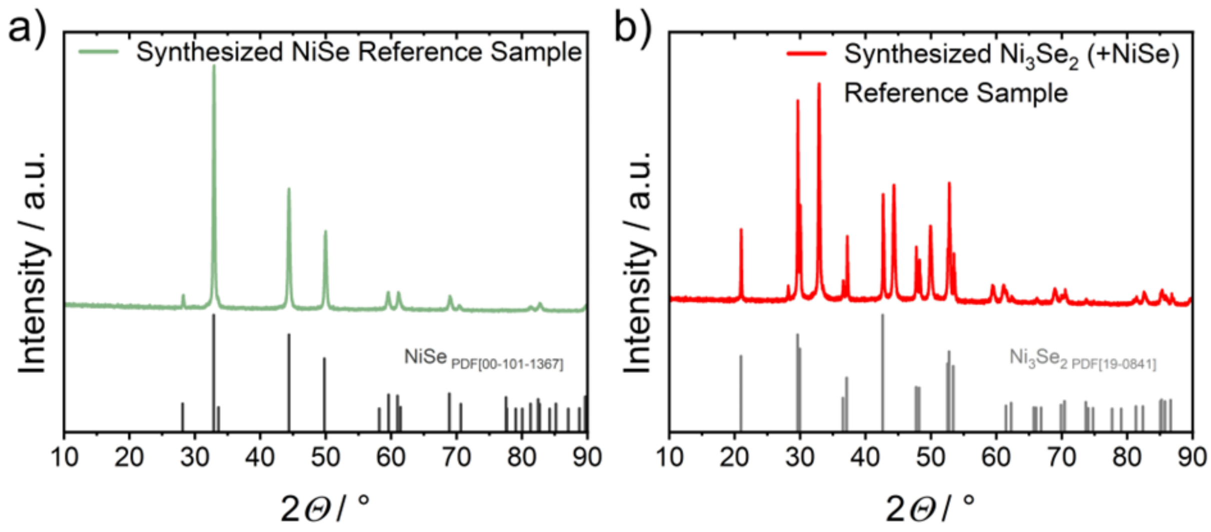

Figure S2. PXRD-pattern of reference materials recorded on Si background of a) synthesized hexagonal NiSe (phase pure) and b) rhombohedral Ni_3Se_2 (main phase, hexagonal NiSe minor phase). The experimentally found signals in both synthesized samples agree with those reported for the respective species' PXRD patterns.

1.5.4 XAS Measurements

Ex situ X-ray absorption near structure (XANES) and extended X-ray absorption fine structure (EXAFS) at Ni K-edge experiments were performed at beamline 20-BM-B of the Advanced Photon Source (APS) of Argonne National Laboratory (ANL). A Vertox ME4 silicon drift diode detector was used to collect the Ni K fluorescence signal while the Si (111) monochromator scanned the incident X-ray photon energy through the Ni K absorption edge. A Ni foil was used as a standard to calibrate the energy. The catalysts were measured on macro electrode substrates (see description in 1.5), while the reference materials were measured in powder form. All XAS data analyses were performed with Athena software package to extract XANES and EXAFS data. The pre-edge was linearly fitted and subtracted. The post-edge background was determined by using a cubic-spline-fit procedure and then subtracted.

The linear combination fitting analysis was done in Athena software package, using the Beer-Lambert law for heterogeneous materials[6] shown in equation (S2):

$$\mu(E) = \sum_{i=1}^{n} w_i \mu_i(E)\,, with \sum_{i} w_i = 1,\ \ w_i \geq 0 \qquad \text{(S2).}$$

There μ is the X-ray absorption coefficient and w is the atomic fraction.

1.5.5 XPS Measurements and detailed Analyses

X-ray photoelectron spectroscopy (XPS) measurements of the nickel selenide films were conducted in an ultra-high vacuum (UHV) setup with a monochromatic Al Kα X-ray source (λ = 1486.6 eV; anode operating at 14.5 kV and 30.5 mA) and a high-resolution Gammadata-Scienta SES 2002 analyzer. XPS measurements of the reference spectra of the NiSe and Ni_3Se_2 powder

samples were performed using a Thermo Fisher ESCALAB 250 Xi setup in UHV conditions with a monochromatic Al Kα X-ray source (λ = 1486.6 eV; anode operating at 14.5 kV and 30.5 mA).To mitigate charge accumulation effects, a flood gun was applied in both setups. All samples were grounded to the sample holder and the take-off angle was 90°. The powder samples were loaded on a sample holder with a recession and pressed with a zirconia tool to yield an even surface. All data analysis was conducted using the software CasaXPS version 2.3.25PR1.0.[7] The binding energy scale of the spectra was charge corrected by setting the C 1s of C-C, C-H of adventitious carbon to 284.8 eV.[8] To subtract the background a Shirley background was applied to the spectra. The lineshapes of the peaks were described in CasaXPS by a Gaussian-Lorentzian GL(m) product formula where m describes the mixing of Lorentzian and Gaussian, with m = 100 being a purely Lorentzian line shape. The lineshape of the metallic Ni 2p peak in CasaXPS was defined using a Lorentzian asymmetric lineshape LA(α,β,m) where m is a value between 0 and 499 describing the width of the Gaussian convoluted with the Lorentzian curve. The spread of the Lorentzian tail is defined by the parameters α (higher binding energy) and β (lower binding energy) with higher values meaning a lower spread.

Based on the fitting of the Ni $2p_{3/2}$, the shape of the Ni LMM, and the O 1s spectra, it can be concluded that the major chemical species in the sample after OER is $Ni(OH)_2$.[9,10] However, small contributions of either γ-NiOOH or β-NiOOH to the Ni 2p spectra alongside $Ni(OH)_2$ cannot be fully excluded due to similarities in their spectral shape.

1.5.5.1 Fitting Procedure

To identify the chemical nature of nickel in the nickel selenide films, an approach based on the work of Biesinger et al. was utilized to fit the Ni $2p_{3/2}$.[10] The peak models for metallic Ni, NiO,

$Ni(OH)_2$, γ-NiOOH, and β-NiOOH of Biesinger et al. were used to fit the spectra by constraining them in their relative area, relative peak position, and relative FWHM (see Table S5 -S7). To account for the variation in adventitious carbon referencing, the position of the model was allowed to vary by ±0.2 eV.[11] The FWHM of the peak components was allowed to increase by up to 0.5 eV from the literature values. To obtain insights into the nickel selenide species on the surface, high resolution spectra of the Ni 2p and Se 3d region of freshly synthesized NiSe and Ni_3Se_2 reference particles were recorded. Empirical peak models of the Ni $2p_{3/2}$ and Se 3d were constructed analogously (see Table S1-S4 and Figures S3+S4). The peak components in the peak models were chosen to obtain appropriate fittings of the reference spectra and are not intended to be interpreted as physical properties of the sample. For more details on peak models, and constraints on the fits and fit results of the different regions refer to Tables S5-S9 in SI-Sections 1.5.5.3 and 1.5.5.4.

To reduce the contribution of surface hydroxides and probe the surface underlying catalyst layers from the pristine nickel selenide film, Ar sputtering was applied. The surface layer of the pristine nickel selenide film was removed by Ar sputtering (1 keV, 5×10^{-5} mbar, 15 min) inside the sputtering chamber. The ion energy was intentionally reduced to minimize reduction effects, ensuring the lowest possible beam energy for oxide surface cleaning and depth profiling.

Due to the low oxygen content after the sputtering, the models of oxygenated Ni compounds were not considered for the fitting of the Ni $2p_{3/2}$. Fitting of the Se 3d revealed that the Se is mostly present as NiSe and a minor amount of Ni_3Se_2 is observed (Table S9, Figure S5). Based on these considerations, the Ni $2p_{3/2}$ was fit with peak models for NiSe and metallic Ni, revealing a significant amount of nickel in metallic state (Table S7, Figure S5), presumably caused by reduction of the surface during the sputtering process.[12] No adventitious carbon could be

identified in the spectra of the Ar-sputtered nickel selenide sample, and only the Se LMM auger structure features were detected. To perform charge correction, the binding energy of the O 1s peak assumed to relate to nickel hydroxide was set to the average binding energy value for $Ni(OH)_2$ of 531.25 ± 0.36 eV (n = 15) extracted from the NIST X-ray Photoelectron Spectroscopy Database (SRD 20), Version 5.0.[13] The position constraints for the peak models used to fit this sample were set to ±0.36 eV.

1.5.5.2 Empirical Fit of the Ni $2p_{3/2}$ and Se 3d of the Reference Samples for NiSe and Ni_3Se_2 and Constructed Reference Models

Table S1. Peak model for the Ni $2p_{3/2}$ of NiSe constructed from the spectrum of the reference material powder with peak positions, FWHM, and area of the peaks.

Species	Peak	Line Shape	Position / eV	FWHM / eV	Area / a.u.
NiSe	1	GL(30)	852.4 *	0.73 *	None
	2	GL(30)	NiSe-(1) + 0.34	NiSe-(1) + 0.96	NiSe-(1) · 1.36024
	3	GL(30)	NiSe-(1) + 2.63	NiSe-(1) + 1.17	NiSe-(1) · 1.68040
	4	GL(30)	NiSe-(1) + 4.67	NiSe-(1) + 1.50	NiSe-(1) · 0.57238
	5	GL(30)	NiSe-(1) + 6.94	NiSe-(1) + 3.27	NiSe-(1) · 0.95434

**For the fitting, NiSe-(1) was constrained to 852.2 – 852.6 eV in position and 0.73 – 1.23 eV in FWHM.*

Table S2. Peak model for the Ni $2p_{3/2}$ of Ni_3Se_2 constructed from the spectrum of the reference material powder with positions, FWHM, and area of the peaks.

Species	Peak	Line Shape	Position / eV	FWHM / eV	Area / a.u.
Ni_3Se_2	1	GL(30)	852.4 *	0.70 *	None
	2	GL(30)	Ni_3Se_2-(1) + 0.24	Ni_3Se_2-(1) + 1.43	Ni_3Se_2-(1) · 1.27558
	3	GL(30)	Ni_3Se_2-(1) + 3.16	Ni_3Se_2-(1) + 1.91	Ni_3Se_2-(1) · 1.78035
	4	GL(30)	Ni_3Se_2-(1) + 6.88	Ni_3Se_2-(1) + 2.87	Ni_3Se_2-(1) · 0.62528
	5	GL(30)	Ni_3Se_2-(1) + 9.37	Ni_3Se_2-(1) + 0.89	Ni_3Se_2-(1) · 0.10869

**For the fitting, Ni_3Se_2-(1) was constrained to 852.2 – 852.6 eV in position and 0.70 – 1.20 eV in FWHM.*

Table S3. Peak model for the Se 3d of NiSe constructed from the spectrum of the reference material powder with peak positions, FWHM, and area of the peaks.

Species	Peak	Line Shape	Position / eV	FWHM / eV	Area / a.u.
NiSe	1	GL(30)	53.7 *	0.55 *	None
	2	GL(30)	NiSe-(1) + 0.88	NiSe-(1) + 0	NiSe-(1) · 0.66202
	3	GL(30)	NiSe-(1) + 0.29	NiSe-(1) + 1.05	NiSe-(1) · 1.51650
	4	GL(30)	NiSe-(1) + 0.85	NiSe-(1) + 1.05	NiSe-(1) · 1.01069
	5	GL(30)	NiSe-(1) + 2.58	NiSe-(1) + 1.95	NiSe-(1) · 0.45560

**For the fitting, NiSe-(1) was constrained to 53.5 – 53.9 eV in position and 0.55 – 1.05 eV in FWHM.*

Table S4. Peak model for the Se 3d of Ni_3Se_2 constructed from the spectrum of the reference material powder with peak positions, FWHM, and area of the peaks.

Species	Peak	Line Shape	Position / eV	FWHM / eV	Area / a.u.
Ni_3Se_2	1	GL(30)	54.4 *	0.40 *	None
	2	GL(30)	Ni_3Se_2-(1) + 0.82	Ni_3Se_2-(1) + 0	Ni_3Se_2-(1) · 0.66598
	3	GL(30)	Ni_3Se_2-(1) – 0.39	Ni_3Se_2-(1) + 0.88	Ni_3Se_2-(1) · 4.50103
	4	GL(30)	Ni_3Se_2-(1) + 0.57	Ni_3Se_2-(1) + 0.88	Ni_3Se_2-(1) · 2.99897
	5	GL(30)	Ni_3Se_2-(1) + 2.13	Ni_3Se_2-(1) + 2.10	Ni_3Se_2-(1) · 1.14433

**For the fitting, Ni_3Se_2-(1) was constrained to 54.2 – 54.6 eV in position and 0.40 – 0.90 eV in FWHM.*

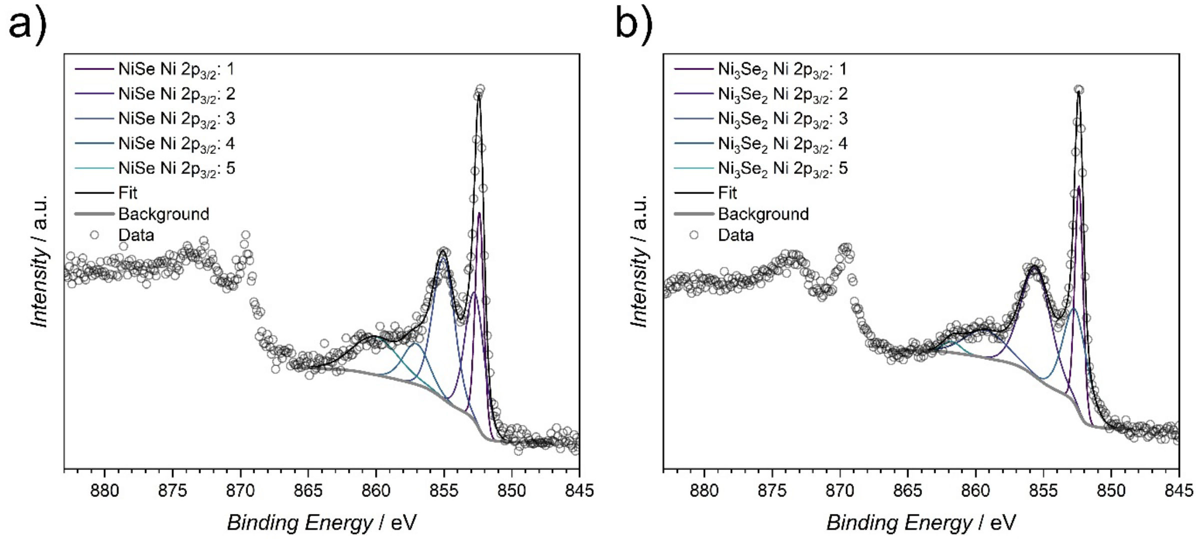

Figure S3. XPS spectra of the Ni $2p_{3/2}$ region of the (a) NiSe and (b) Ni_3Se_2 reference materials were recorded at a pass energy of 10 eV with peaks of the empirical peak models that were used to fit the electrodeposited samples.

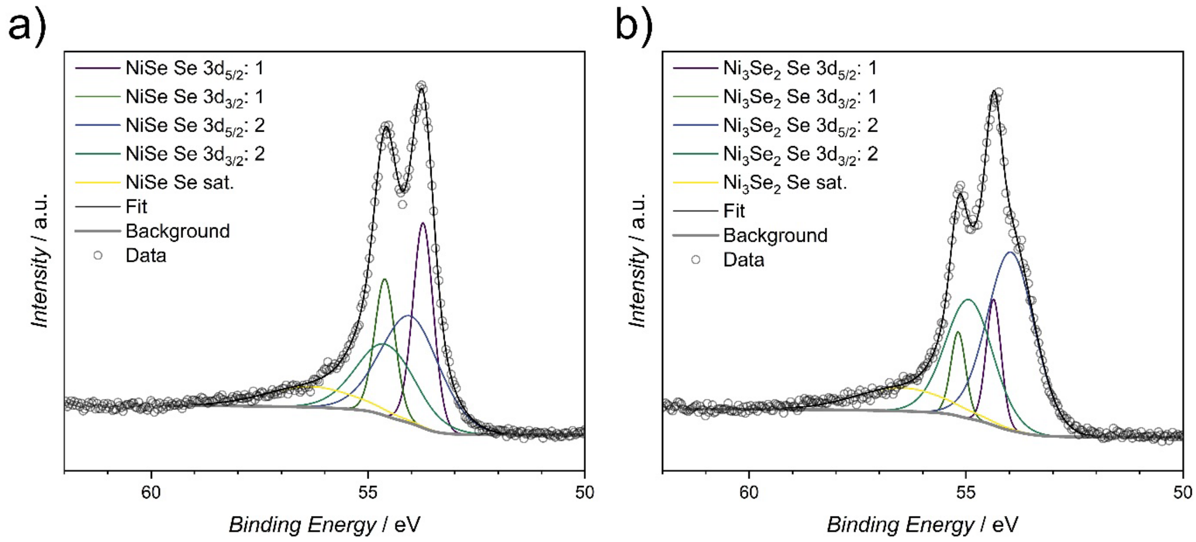

Figure S4. XPS spectra of the Se 3d region of the (a) NiSe and (b) Ni_3Se_2 reference materials were recorded at a pass energy of 10 eV with peaks of the empirical peak models that were used to fit the electrodeposited samples.

1.5.5.3 Details of the Peak Fitting of the Nickel Selenide Samples

Table S5. Details of the fit of the Ni $2p_{3/2}$ region of as-deposited nickel selenide film based on reference models. The peak positions, FWHM, and area of the peak components in % are listed together with concentrations of the species in %.

Species	Peak	Line Shape	Position / eV	FWHM / eV	Area / %	Species Conc. / %
NiSe	1	GL(30)	852.5	0.81	11.7	65
	2	GL(30)	852.9	1.76	16.0	
	3	GL(30)	855.2	1.97	19.7	
	4	GL(30)	857.2	2.31	6.7	
	5	GL(30)	859.5	4.07	11.2	
$Ni(OH)_2$	1	GL(30)	855.1	1.43	2.6	35
	2	GL(30)	855.9	2.56	15.7	
	3	GL(30)	857.9	1.86	1.0	
	4	GL(30)	860.7	1.33	0.5	
	5	GL(30)	861.7	4.92	13.6	
	6	GL(30)	866.6	3.28	1.3	

Table S6. Details of the fit of the Ni $2p_{3/2}$ region of the nickel selenide film after OER based on reference models. The peak positions, FWHM, and area of the peak components in % are listed together with concentrations of the species in %.

Species	Peak	Line Shape	Position / eV	FWHM / eV	Area / %	Species Conc. / %
$Ni(OH)_2$	1	GL(30)	855.1	1.66	7.4	100
	2	GL(30)	855.9	2.79	45.3	
	3	GL(30)	857.9	2.09	3.0	
	4	GL(30)	860.7	1.56	1.4	
	5	GL(30)	861.7	5.15	39.2	
	6	GL(30)	866.6	3.51	3.7	

Table S7. Details of the fit of the Ni $2p_{3/2}$ region of as-deposited nickel selenide film based on reference models. The peak positions, FWHM, and area of the peak components in % are listed together with concentrations of the species in %.

Species	Peak	Line Shape	Position / eV	FWHM / eV	Area / %	Species Conc. / %
NiSe	1	GL(30)	852.5	0.73	11.1	62
	2	GL(30)	852.9	1.69	15.0	
	3	GL(30)	855.2	1.90	18.6	
	4	GL(30)	857.2	2.23	6.3	
	5	GL(30)	859.5	4.00	10.6	
Ni(0)	1	LA(1.1,2.2,10)	853.0	1.45	31.3	38
	2	GL(30)	856.6	3.20	2.4	
	3	GL(30)	859.0	3.20	4.8	

1.5.5.4 Peak Fitting Details for the Se 3d

The SeO_x species in the Se 3d XP spectrum of the pristine sample was fitted with only one component, as the splitting of the Se $3d_{3/2}$ and Se 3 $d_{5/2}$ could not be resolved. Further, several SeO_x species may coexist on the surface and overlap in the spectral region.

Table S8. Details of the fit of the Se 3d region of as-deposited nickel selenide film based on reference models. The peak positions, FWHM, and area of the peak components in % are listed together with concentrations of the species in %.

Species	Peak	Line Shape	Position / eV	FWHM / eV	Area / %	Species Conc. / %
NiSe	1	GL(30)	53.9	0.65	20.0	93
	2	GL(30)	54.8	0.65	13.2	
	3	GL(30)	54.2	1.70	30.3	
	4	GL(30)	54.8	1.70	20.2	
	5	GL(30)	56.5	2.60	9.1	
SeO_X	1	GL(30)	58.9	1.84	7.3	7

Table S9. Details of the fit of the Se 3d region of as-deposited nickel selenide film after Ar-sputtering based on reference models. The peak positions, FWHM, and area of the peak components in % are listed together with concentrations of the species in %.

Species	Peak	Line Shape	Position / eV	FWHM / eV	Area / %	Species Conc. / %
NiSe	1	GL(30)	54.0	0.66	19.2	89
	2	GL(30)	54.9	0.66	12.7	
	3	GL(30)	54.3	1.71	29.2	
	4	GL(30)	54.8	1.71	19.4	
	5	GL(30)	56.6	2.61	8.8	
Ni_3Se_2	1	GL(30)	54.5	0.42	1.0	11
	2	GL(30)	55.3	0.42	0.7	
	3	GL(30)	54.1	1.30	4.7	
	4	GL(30)	55.1	1.30	3.1	
	5	GL(30)	56.6	2.52	1.2	

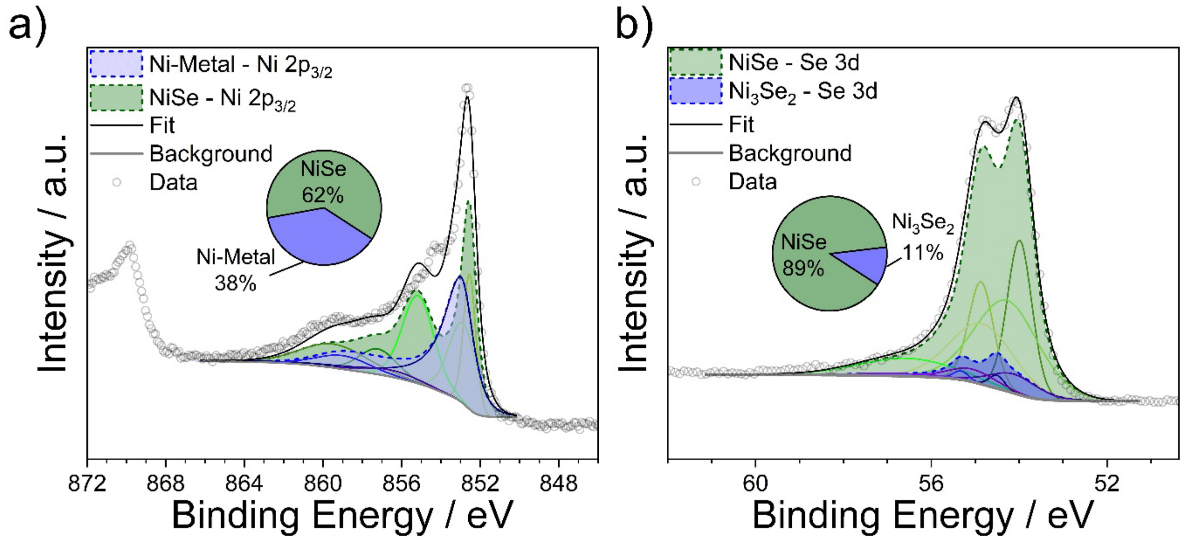

Figure S5.XPS-spectra of the (a) Ni $2p_{3/2}$ and (b) Se 3d region of an as-deposited sample after Ar-sputtering.

1.5.5.5 Auger Peaks of Nickel

The significant changes in the Ni LMM auger signal support the conclusion that the chemical nature of the nickel in the surface-near region changed after OER. According to literature, a feature at higher binding energy next to the main auger peak would be expected for γ-NiOOH. Hence, the Ni LMM of the sample after OER helps us support our claim that the nickel near the surface exists as a $Ni(OH)_2$-like species and not as γ-NiOOH.[14]

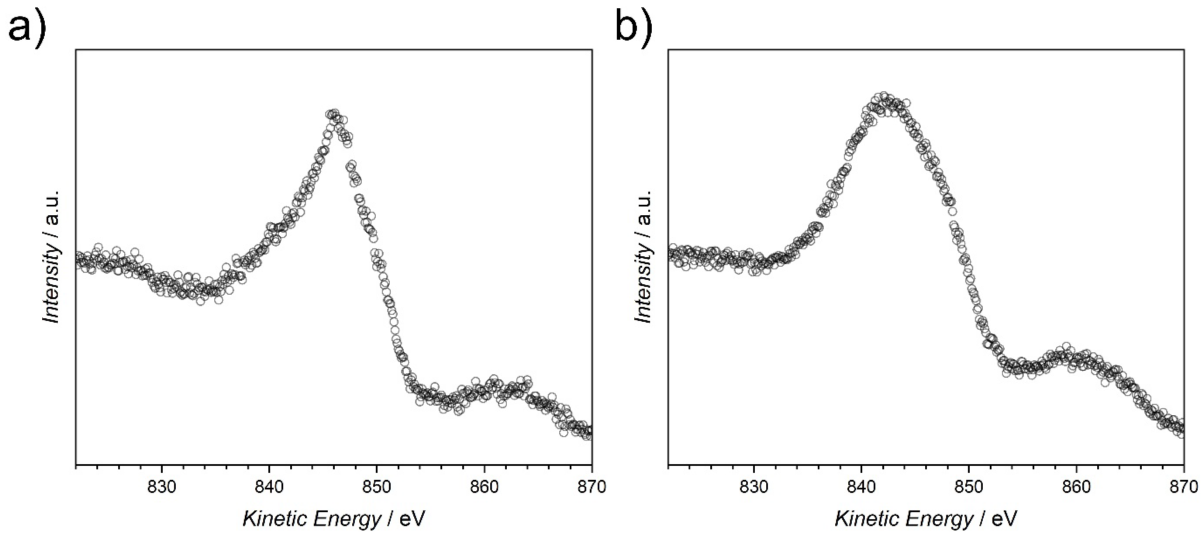

Figure S6. XPS-spectra of the Ni LMM auger signal of (a) the pristine nickel selenide film and (b) a nickel selenide film after OER.

1.5.5.6 Peak Fitting Details of the O 1s

Table S10. Details of the fit of the O 1s region of the nickel selenide samples. The peak positions, FWHM, the constraints on the FWHM, and the area of the peak components in % are listed together.

As-deposited Nickel selenide film						
Species	Peak	Line Shape	Position / eV	FWHM / eV	FWHM Constr. / %	Area / %
Hydroxides	A	GL(30)	531.2	1.61	0.5 , 2.0	86
Ads. Water, Organics	B	GL(30)	532.8	1.61	A · 1	14
**Nickel selenide film after OER**						
Species	Peak	Line Shape	Position / eV	FWHM / eV	FWHM Constr. / %	Area / %
Hydroxides	A	GL(30)	530.8	1.77	0.5 , 2.0	69
Ads. Water, Organics	B	GL(30)	532.2	1.77	A · 1	31
**As-deposited Nickel selenide film after Ar-Sputtering**						
Species	Peak	Line Shape	Position / eV	FWHM / eV	FWHM Constr. / %	Area / %
Lattice O	A	GL(30)	529.1	0.99	0.5 , 1.5	13
Hydroxides	B	GL(30)	531.25*	1.75	0.5 , 2.0	70
Ads. Water, Organics	C	GL(30)	533.4	1.75	B · 1	18

**The position of this peak was set to 531.25 eV to calibrate the binding energy axis.*

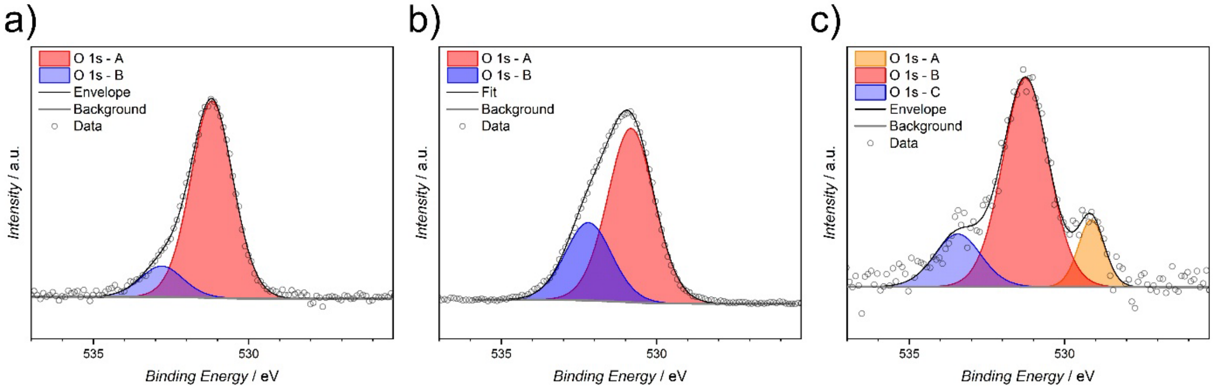

Figure S7. XPS-spectra of the O 1s region of (a) the as-deposited nickel selenide, (b) the nickel selenide film after OER, and (c) an as-deposited sample after Ar-sputtering.

The peak labeled A in the O 1s of the as-deposited nickel selenide film and the nickel selenide film after OER could be related to the hydroxide species in nickel hydroxides.[15] For the as-deposited sample, a contribution of the oxygenated selenium species that were identified in the Se 3d region is expected but was not assigned due to the lack of literature data. The peak labeled B in the O 1s could be attributed to adsorbed water, hydrated $Ni(OH)_2$, or oxygenated organic contaminations.[15,16] The O 1s spectrum of the sample after OER further supports the major presence of $Ni(OH)_2$ as concluded from the analysis of the Ni $2p_{3/2}$. If significant amounts of β- or γ-NiOOH were present on the sample surface, a peak at 529.3-529.5 eV would be expected which has not been observed.[17]

The small oxygen remaining O 1s peaks in the Ar-sputtered samples may be related to NiO (peak A), $Ni(OH)_2$ (peak B), and adsorbed water (peak C) respectively. Due to the absence of a Se-O peak at ~58-60 eV, contributions from selenium oxygen components can be excluded.

1.5.5.7 C 1s Region of the XPS Spectra

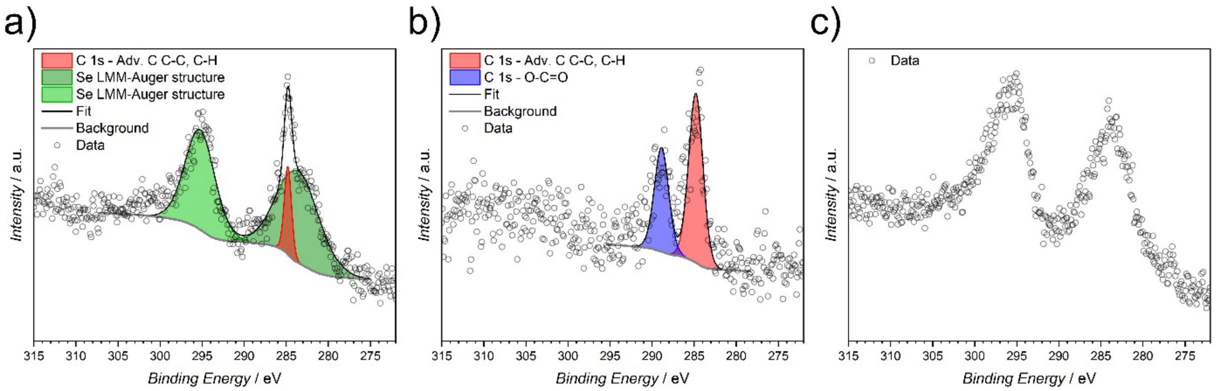

Figure S8. XPS-spectra of the C 1s region of (a) the as-deposited nickel selenide film, (b) the nickel selenide film after OER, and (c) an as-deposited sample after Ar-sputtering. For the as-deposited nickel selenide film, the Se LMM auger structure overlaps with the C 1s peak. For the Ar-sputtered sample, no C1s peak was identified due to the intense contributions of the Se LMM auger structure.

Table S11. Details of the fit of the C 1s region of the nickel selenide samples. The peak positions, FWHM, the constraints on the FWHM, and the area of the peak components in % are listed together.

As-deposited Nickel selenide film						
Species	Peak	Line Shape	Position / eV	FWHM / eV	FWHM Constr. / %	Area / %
C 1s, Adv. C, C-C, C-H	A	GL(30)	284.8*	1.01	0.35 , 2	N.A.
Se Auger	B	GL(30)	283.6	5.80	1 , 10	N.A.
Se Auger	C	GL(30)	295.2	4.19	1, 10	N.A.
**Nickel selenide film after OER**						
Species	Peak	Line Shape	Position / eV	FWHM / eV	FWHM Constr. / %	Area / %
C 1s, Adv. C, C-C, C-H	A	GL(30)	284.8*	2.00	0.35 , 2	62
C 1s, Adv. C, O-C=O	B	GL(30)	288.9	2.00	A · 1	38

**The position of this peak was set to 284.8 eV to calibrate the binding energy axis.*

1.5.5.8 Survey Scans of the Electrodeposited Nickel Selenide Films

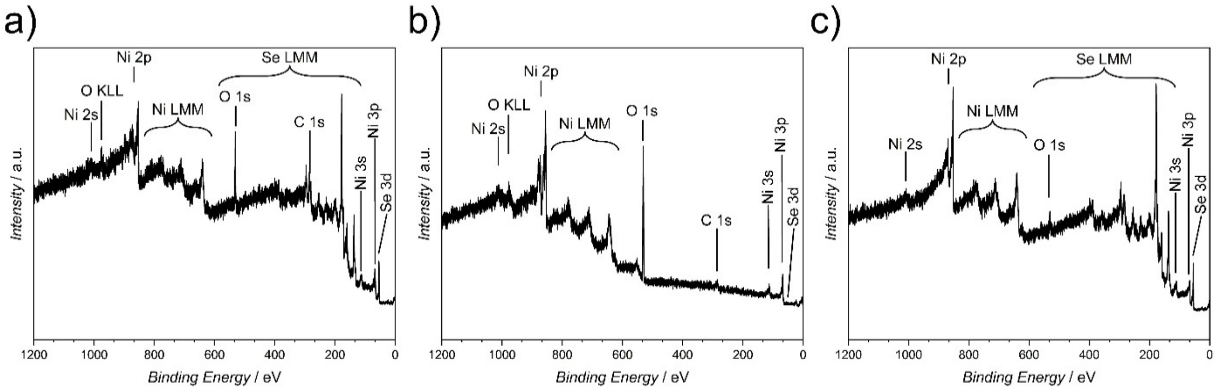

Figure S9. XPS-survey spectra recorded at pass energy of 200 eV of (a) the as-deposited nickel selenide film (pristine sample), (b) the nickel selenide film after OER (after OER sample), and (c) an as-deposited sample after Ar-sputtering (Ar-sputtered pristine sample).

Table S12. Surface composition calculated by integrating the Ni 3p, O 1s and Se 3d region from the survey spectra of the as-deposited sample, the nickel selenide film after OER, and an as-prepared sample after Ar sputtering.

Sample	At. % Nickel	At. % Selenium	At. % Oxygen
Pristine	39	26	35
After OER	30	<1	70
Ar-sputtered Pristine	53	42	5

2. Additional Data

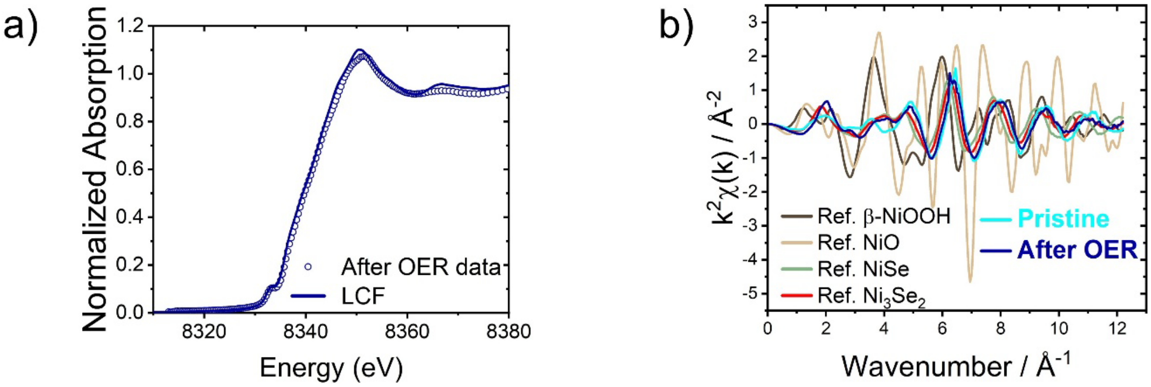

Figure S10. a) Linear combination fitting (LCF) of the after OER EXAFS data. The fit reflects a NiO share of ≈ 20% (≈ 80% of pristine nickel selenide species); b) Ni K-edge EXAFS spectra in k-space of pristine and after OER samples.

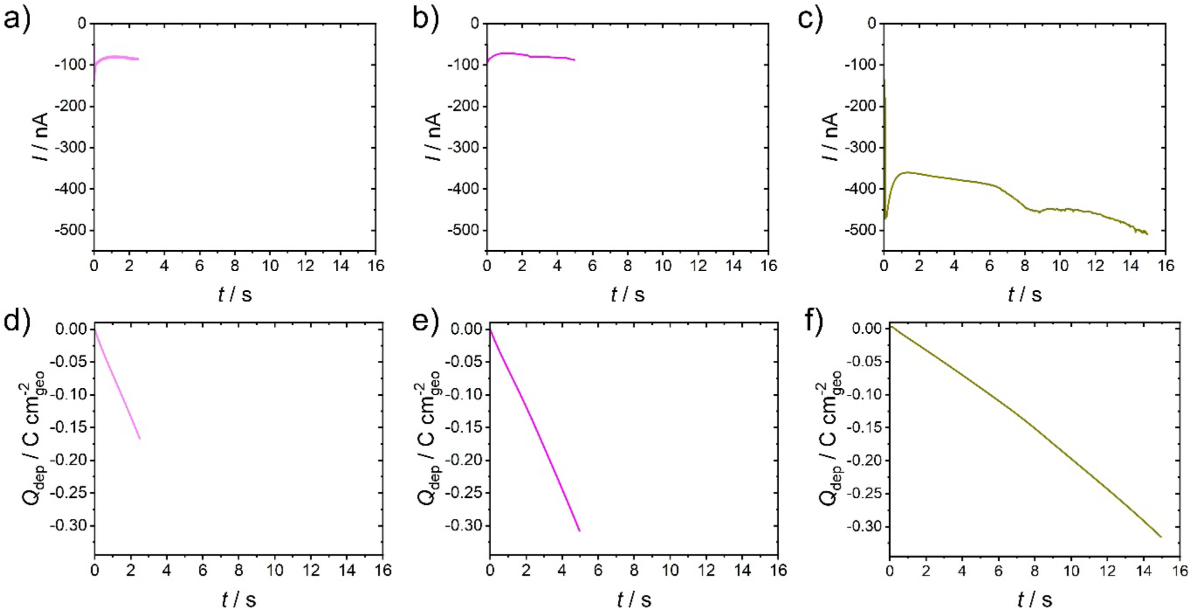

Figure S11. Chronoamperometric and coulometric deposition curves of nickel selenide deposited from a solution of 10 mM $NiSO_4$, 10 mM SeO_2, and 25 mM K_2SO_4 (pH 2.5) at −0.79 V vs. Ag|AgCl|3 M KCl on a), d) 12.7 µm (0.16 C cm^{-2}), b), e) 12.7 µm (0.32 C cm^{-2}) and c), f) 50.0 µm (0.32 C cm^{-2}) UMEs.

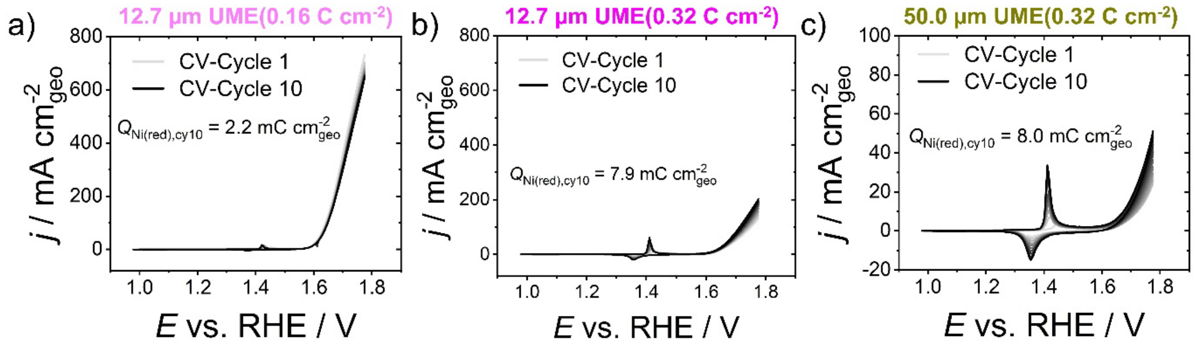

Figure S12. Cyclic voltammograms of the 10-CV-cycle-activation periods of nickel selenide in 0.1 M KOH + 1 mM $Fe(NO_3)_3$ in a potential window of 0.98 – 1.78 V vs. RHE at a scan rate of 100 mV s^{-1} electrodeposited on a) 12.7 µm (0.16 C cm^{-2}), b) 12.7 µm (0.32 C cm^{-2}) and c) 50.0 µm (0.32 C cm^{-2}) UMEs. In the case of a) 12.7 µm (0.16 C cm^{-2}) UME, the activity decreased from cycle 1 to cycle 10, which indicates a starting deactivation under mass-transport-controlled conditions. On the other hand, in the two experiments with doubled charge loading on b) 12.7 µm (0.32 C cm^{-2}) and c) 50.0 µm (0.32 C cm^{-2}) UMEs, the activity increased from cycle 1 to cycle 10, indicating an ongoing activation of the catalyst material. Noteworthy, the share of electrochemically active nickel extracted in each of the three CV activation experiments from the 10th CV-cycle by integrating the nickel reduction peak ($Q_{Ni(red),cy10}$) is more than 3.5 times higher for a) lower catalyst loading ($Q_{Ni(red),cy10}$ = 2.2 mC cm^{-2}) compared to the higher loadings on both, b) 12.7 µm ($Q_{Ni(red),cy10}$ = 7.9 mC cm^{-2}) and c) 50.0 µm ($Q_{Ni(red),cy10}$ = 8.0 mC cm^{-2}) UMEs. The similar $Q_{Ni(red),cy10}$ for the 0.32 C cm^{-2} charge loading ensures high consistency between the experiments on UMEs of both sizes.

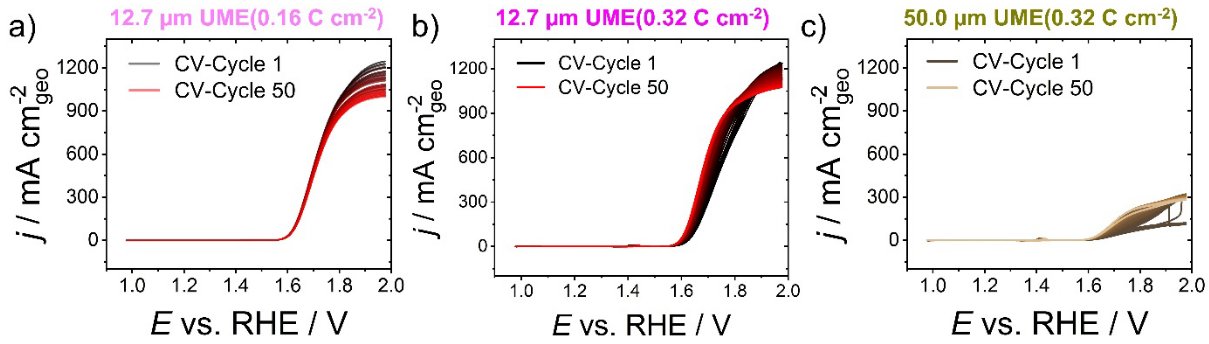

Figure S13. Cyclic voltammograms of the 50-CV-cycle-characterization periods of nickel selenide in 0.1 M KOH + 1 mM $Fe(NO_3)_3$ in a potential window of 0.98 – 1.98 V vs. RHE at a scan rate of 10 mV s^{-1} electrodeposited on a) 12.7 µm (0.16 C cm^{-2}), b) 12.7 µm (0.32 C cm^{-2}) and c) 50.0 µm (0.32 C cm^{-2}) UMEs. In the case of a) 12.7 µm (0.16 C cm^{-2}), the activity decreased from cycle 1 to cycle 50, in both, kinetic and mass-transport-controlled overpotential regions, indicating degradation of the catalytically active species. On the other hand, in experiment b) 12.7 µm (0.32 C cm^{-2}), the activity increased from cycle 1 to cycle 50, in the kinetically controlled potential region, while it decreased at high overpotentials under conditions dominated by mass transport. These findings indicate an ongoing activation of the catalyst material (kinetic region), while the catalytic activity under high overpotentials is hampered (mass-transport-controlled region). For the third experiment on c) 50.0 µm (0.32 C cm^{-2}) UME, a kinetic activation indicated by an increase in current density in the lower overpotential range from CV-cycle 1 to 50 can be observed, while also the current response (and with this activity) at high overpotentials increases in the first CV-cycles. With ongoing CV cycling, mass-transport phenomena seem to limit the activity of the catalytically active species at high overpotentials, resulting in a current density decrease. Noteworthy, the current densities at the highest overpotentials recorded on the larger UME in c) 50.0 µm (0.32 C cm^{-2}) are about four times smaller than those recorded on the smaller UMEs in a) 12.7 µm (0.16 C cm^{-2}) and b) 12.7 µm

(0.32 C cm^{-2}). The fact that the recorded current densities in experiments a) and b) are similar, despite the doubled charge loading in b), supports the assumption of mass-transport-limitation.

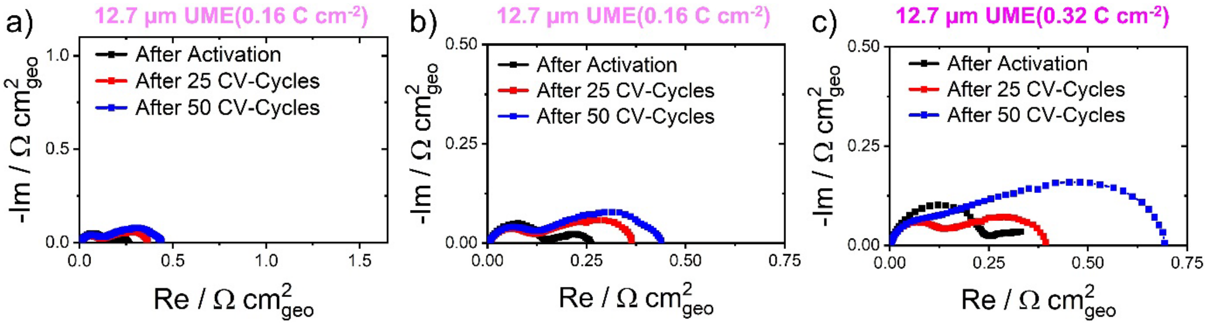

Figure S14. Nyquist plots of EIS spectra recorded at 1.8 V vs. RHE **after activation**, **after 25 CV cycles** and **50 CV cycles** of OER characterization for a) + b) 12.7 µm (0.16 C cm^{-2}) (a) full range, b) zoomed in) and c) 12.7 µm (0.32 C cm^{-2}) UMEs (only zoomed in, full range shown in Figure 5b) of the main article).

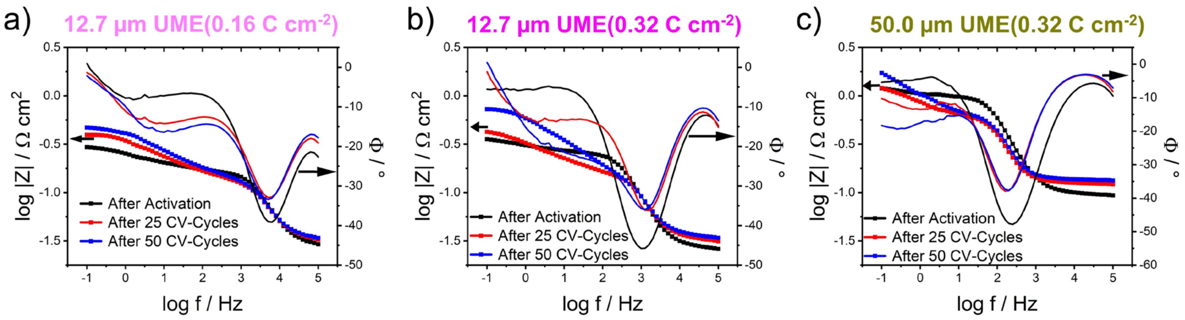

Figure S15. Bode plots of EIS spectra recorded at 1.8 V vs. RHE **after activation**, **after 25 CV cycles** and **50 CV cycles** of OER characterization on a) 12.7 µm (0.16 C cm^{-2}), b) 12.7 µm (0.32 C cm^{-2}) and c) 50.0 µm (0.32 C cm^{-2}) UMEs.

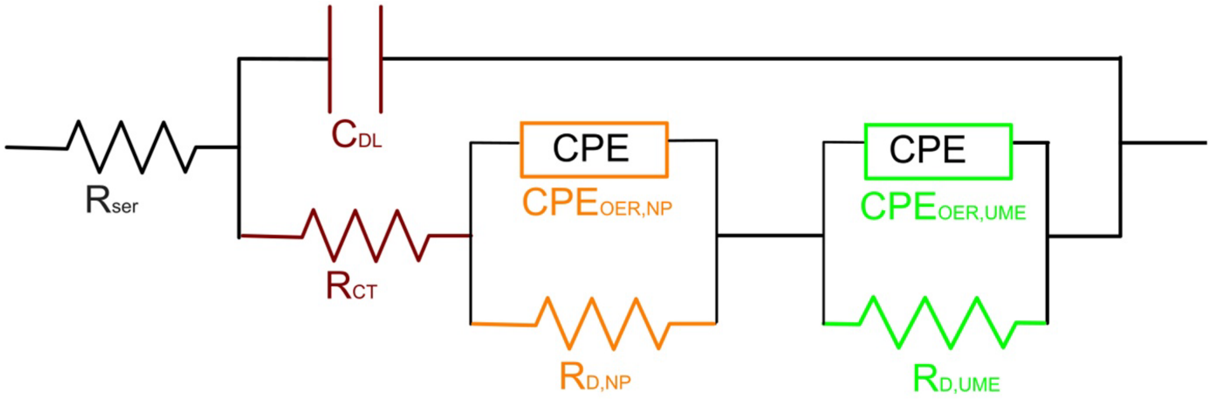

Figure S16. The equivalent circuit used to fit the experimental EIS spectra after 50 CV-cycles on 12.7 µm (0.32 C cm^{-2}) and 50.0 µm (0.32 C cm^{-2}) UMEs (presented in the main article Figure 6). The equivalent circuit) consists of a serial resistor (Rser, subtracted for normalized data), an R-C element representing the charge transfer from the NPs to the electrode (**R_{CT}** and **C_{CT}**) as well as two R-CPE elements representing the mass transport under OER conditions to the single NPs (**$R_{D,NP}$** and **$CPE_{D,NP}$**) and the total UME (**$R_{D,UME}$** and **$CPE_{D,UME}$**), respectively.

Table S13. Parameters extracted from fitting the equivalent circuit shown in Figure S16 to the experimental EIS spectra recorded after 50 CV cycles at 1.8 V vs. RHE on 12.7 μm (0.16 C cm^{-2} and 0.32 C cm^{-2} catalyst loadings) and 50.0 μm (0.32 C cm^{-2} catalyst loading).

	**12.7 μm UME**		**50.0 μm UME**
	0.16 C cm^{-2}	0.32 C cm^{-2}	0.32 C cm^{-2}
	Value ± Error	Value ± Error	Value ± Error
R_{ser} / kΩ	28.30 ± 0.55	29.42 ± 0.47	7.18 ± 0.14
$\boldsymbol{R_{CT}}$ **/ Ω cm²**	0.078 ± 0.006	0.096 ± 0.006	0.453 ± 0.008
$\boldsymbol{R_{D,NP}}$ **/ Ω cm²**	0.141 ± 0.073	0.377 ± 0.078	0.112 ± 0.093
$\boldsymbol{R_{D,UME}}$ **/ Ω cm²**	0.230 ± 0.063	0.223 ± 0.067	2.46 ± 0.25
$\boldsymbol{C_{CT}}$ **/ mF cm^{-2}**	0.534 ± 0.001	1.748 ± 0.051	3.391 ± 0.068
**CPE $Q_{D,NP}$ / mF cm^{-2}**	582 ± 277	228 ± 48	727 ± 133
**CPE $\alpha_{D,NP}$**	0.435 ± 0.072	0.579 ± 0.041	0.80 ± 0.18
**CPE $Q_{D,UME}$ / mF cm^{-2}**	593 ± 106	711 ± 149	779 ± 81
**CPE $\alpha_{D,UME}$**	0.702 ± 0.047	0.947 ± 0.071	0.542 ± 0.039

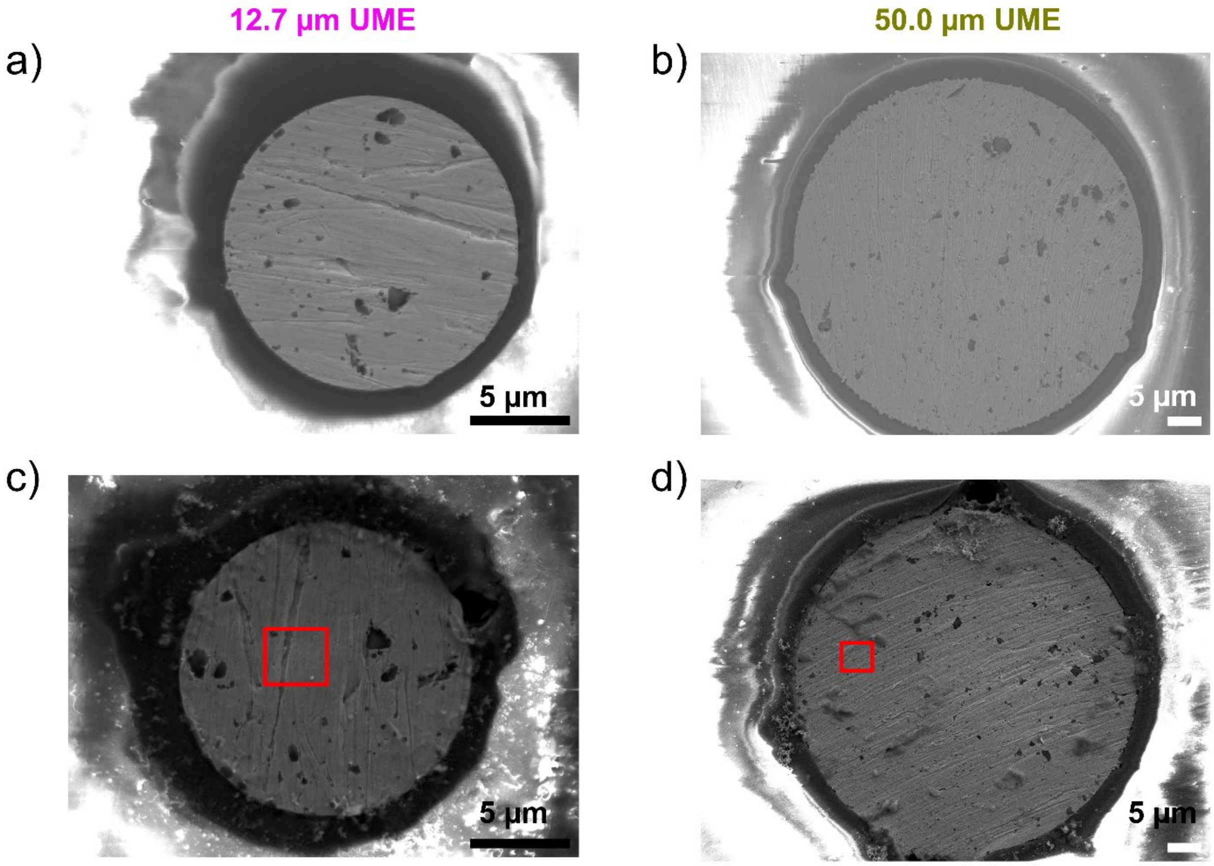

Figure S17. Overview SEM images recorded at 10 kV of the total areas of 12.7 µm and 50.0 µm UMEs a) + b) before and c) + d) after deposition of nickel selenides (charge loading 0.32 C cm^{-2}). The areas in which the identical location (IL) SEM measurements were conducted are indicated by red rectangles.

Table 14. Table of atomic shares extracted of the identified elements of deposited catalyst materials (charge loading 0.32 C cm^{-2}) on 12.7 μm UME in a) pristine and b) after OER states as well as on 50.0 μm UME in c) pristine and d) after OER states in the EDX spectra in Figures 7 and 8 of the main manuscript.

Map Sum Spectrum – 12.7 μm UME Pristine		Map Sum Spectrum – 12.7 μm UME After OER		Map Sum Spectrum – 50.0 μm UME Pristine		Map Sum Spectrum – 50.0 μm UME After OER	
Element	Atomic %	Element	Atomic %	Element	Atomic %	Element	Atomic %
C	37	C	36	C	31	C	33
O	3	O	14	O	10	O	22
Al	0	Al	1	Al	2	Al	2
Fe	0	Fe	0	Fe	0	Fe	1
Ni	9	Ni	6	Ni	8	Ni	6
Se	6	Se	0	Se	5	Se	0
Au	45	Au	43	Au	44	Au	36
Total:	100	Total:	100	Total:	100	Total:	100

REFERENCES

(1) Oesch, U.; Janata, J. Electrochemical Study of Gold Electrodes with Anodic Oxide Films—I. Formation and Reduction Behaviour of Anodic Oxides on Gold. Electrochimica Acta 1983, 28 (9), 1237–1246. DOI: 10.1016/0013-4686(83)85011-7.

(2) Cao, X.; Hong, Y.; Zhang, N.; Chen, Q.; Masud, J.; Zaeem, M. A.; Nath, M. Phase Exploration and Identification of Multinary Transition-Metal Selenides as High-Efficiency Oxygen Evolution Electrocatalysts through Combinatorial Electrodeposition. ACS Catal. 2018, 8 (9), 8273–8289. DOI: 10.1021/acscatal.8b01977.

(3) Trotochaud, L.; Young, S. L.; Ranney, J. K.; Boettcher, S. W. Nickel-Iron Oxyhydroxide Oxygen-Evolution Electrocatalysts: The Role of Intentional and Incidental Iron Incorporation. Journal of the American Chemical Society 2014, 136 (18), 6744–6753. DOI: 10.1021/ja502379c.

(4) Bard, A. J.; Faulkner, L. R.; White, H. S. Electrochemical Methods:Fundamentals and Applications, Third edition; Wiley, 2022.

(5) Yuan, B.; Luan, W.; Tu, S. One-Step Solvothermal Synthesis of Nickel Selenide Series: Composition and Morphology Control. CrystEngComm 2012, 14 (6), 2145. DOI: 10.1039/c2ce06474j.

(6) Chantler, C. T.; Bunker, G.; D'Angelo, P.; Diaz-Moreno, S. X-ray Absorption Spectroscopy. Nat Rev Methods Primers 2024, 4 (1). DOI: 10.1038/s43586-024-00366-8.

(7) Fairley, N.; Fernandez, V.; Richard-Plouet, M.; Guillot-Deudon, C.; Walton, J.; Smith, E.; Flahaut, D.; Greiner, M.; Biesinger, M.; Tougaard, S.; Morgan, D.; Baltrusaitis, J. Systematic and Collaborative Approach to Problem Solving Using X-Ray Photoelectron Spectroscopy. Applied Surface Science Advances 2021, 5, 100112. DOI: 10.1016/j.apsadv.2021.100112.

(8) Morgan, D. J. The Utility of Adventitious Carbon for Charge Correction: A Perspective From a Second Multiuser Facility. Surf. Interface Anal. 2025, 57 (1), 28–35. DOI: 10.1002/sia.7360.

(9) Grosvenor, A. P.; Biesinger, M. C.; Smart, R. S.; McIntyre, N. S. New Interpretations of XPS Spectra of Nickel Metal and Oxides. Surface Science 2006, 600 (9), 1771–1779. DOI: 10.1016/j.susc.2006.01.041.

(10) Biesinger, M. C.; Payne, B. P.; Lau, L. W. M.; Gerson, A.; St. Smart, R. C. X-Ray Photoelectron Spectroscopic Chemical State Quantification of Mixed Nickel Metal, Oxide and Hydroxide Systems. Surf. Interface Anal. 2009, 41 (4), 324–332. DOI: 10.1002/sia.3026.

(11) Barr, T. L.; Seal, S. Nature of the Use of Adventitious Carbon as a Binding Energy Standard. Journal of Vacuum Science & Technology A: Vacuum, Surfaces, and Films 1995, 13 (3), 1239–1246. DOI: 10.1116/1.579868.

(12) González-Elipe, A. R.; Alvarez, R.; Holgado, J. P.; Espinos, J. P.; Munuera, G.; Sanz, J. M. An XPS Study of the Ar+-Induced Reduction of Ni2+ in NiO and Ni-Si Oxide Systems. Applied Surface Science 1991, 51 (1-2), 19–26. DOI: 10.1016/0169-4332(91)90058-R.

(13) Wagner, C. D.; Naumkin, A. V.; Kraut-Vass, A.; Allison, J. W.; Powell, C. J.; Rumble, J. R., JR. NIST X-ray Photoelectron Spectroscopy Database (SRD 20), Version 5.0 (Web Version) (https://srdata.nist.gov/xps/),

(14) Biesinger, M. C.; Lau, L. W. M.; Gerson, A. R.; Smart, R. S. C. The Role of the Auger Parameter in XPS Studies of Nickel Metal, Halides and Oxides. Physical chemistry chemical physics : PCCP 2012, 14 (7), 2434–2442. DOI: 10.1039/c2cp22419d.

(15) Biesinger, M. C.; Payne, B. P.; Grosvenor, A. P.; Lau, L. W.; Gerson, A. R.; Smart, R. S. Resolving Surface Chemical States in XPS Analysis of First Row Transition Metals, Oxides and Hydroxides: Cr, Mn, Fe, Co and Ni. Applied Surface Science 2011, 257 (7), 2717–2730. DOI: 10.1016/j.apsusc.2010.10.051.

(16) Mansour, A. N.; Melendres, C. A. Characterization of α -Ni(OH)2 by XPS. Surface Science Spectra 1994, 3 (3), 255–262. DOI: 10.1116/1.1247754.

(17) Payne, B. P.; Biesinger, M. C.; McIntyre, N. S. The Study of Polycrystalline Nickel Metal Oxidation by Water Vapour. Journal of Electron Spectroscopy and Related Phenomena 2009, 175 (1-3), 55–65. DOI: 10.1016/j.elspec.2009.07.006.
